# Comparison of small-sample standard-error corrections for generalised estimating equations in stepped wedge cluster randomised trials with a binary outcome: A simulation study

**DOI:** 10.1177/0962280220958735

**Published:** 2020-09-24

**Authors:** JA Thompson, K Hemming, A Forbes, K Fielding, R Hayes

**Affiliations:** 1 Department of Infectious Disease Epidemiology, London School of Hygiene & Tropical Medicine, London, UK; 2 Institute of Applied Health Research, University of Birmingham, Birmingham, UK; 3 Biostatistics Unit, Monash University, Melbourne, Australia

**Keywords:** Stepped wedge cluster randomised trials, correlated data, sandwich variance, small sample corrections, degrees of freedom, generalised estimating equations

## Abstract

Generalised estimating equations with the sandwich standard-error estimator provide a promising method of analysis for stepped wedge cluster randomised trials. However, they have inflated type-one error when used with a small number of clusters, which is common for stepped wedge cluster randomised trials. We present a large simulation study of binary outcomes comparing bias-corrected standard errors from Fay and Graubard; Mancl and DeRouen; Kauermann and Carroll; Morel, Bokossa, and Neerchal; and Mackinnon and White with an independent and exchangeable working correlation matrix. We constructed 95% confidence intervals using a *t*-distribution with degrees of freedom including clusters minus parameters (DF_C-P_), cluster periods minus parameters, and estimators from Fay and Graubard (DF_FG_), and Pan and Wall. Fay and Graubard and an approximation to Kauermann and Carroll (with simpler matrix inversion) were unbiased in a wide range of scenarios with an independent working correlation matrix and more than 12 clusters. They gave confidence intervals with close to 95% coverage with DF_FG_ with 12 or more clusters, and DF_C-P_ with 18 or more clusters. Both standard errors were conservative with fewer clusters. With an exchangeable working correlation matrix, approximated Kauermann and Carroll and Fay and Graubard had a small degree of under-coverage.

## 1. Introduction

Many small sample corrections are available for generalised estimating equations^1^ (GEE) with the sandwich standard-error estimator,^2–7^ but there has been limited research on which are appropriate for use in stepped wedge trials (SW-CRTs) with a binary outcome.

**Figure 1.**
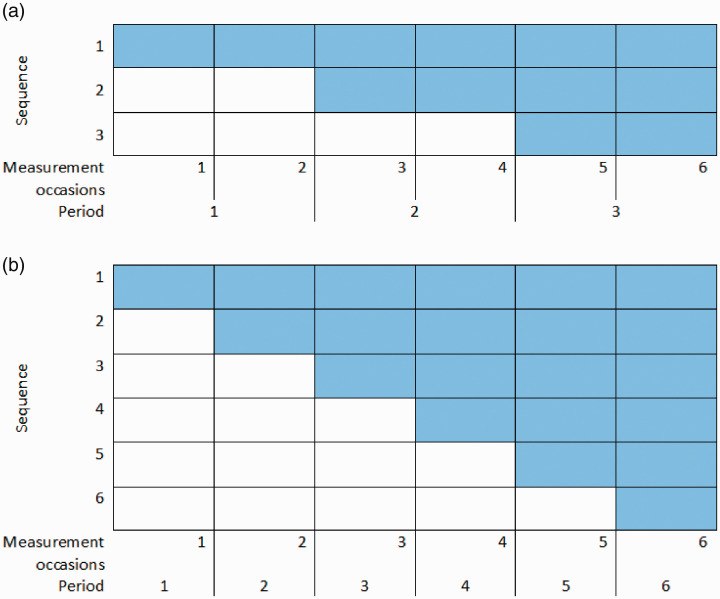
Trial designs used in the simulation study. SW-CRTs with no baseline period and (a) three or (b) six sequences. Coloured cell = intervention, white cells = control.

An SW-CRT is a type of cluster randomised trial (CRT). Groups of individuals, known as clusters, are randomised to different sequences. The sequence dictates the time at which clusters will switch from a control condition to receive the intervention (Figure 1). In practice, SW-CRTS often collect binary outcomes and randomise a small number of clusters: a recent systematic review indicated that 75% of trials randomised 23 or fewer clusters.^8^

Analysis of SW-CRTs requires consideration of the correlation between observations in each cluster. The strength of correlation may depend on the timing of observations within clusters, for example correlation can be stronger between observations from the same cluster collected closer together in time.^9^ GEE, which estimate population average effects, together with the sandwich standard-error estimator account for this correlation and are asymptotically robust to misspecification of correlation structures.^1,10^ This is useful in a trial setting, where analysis methods must be prespecified often with little information to guide the choice of correlation structure.

With a small number of clusters, the standard-error estimates from GEE with sandwich standard-errors are biased, and the variability in the estimated standard errors means than confidence intervals are too narrow when constructed using a normal distribution.^11^ A rule of thumb for parallel CRTs is that 40 clusters are required to avoid these issues,^12^ and this limits the utility of GEE for CRTs.

Many corrections have been developed to improve estimation of sandwich standard-errors in finite samples,^2–5,7^ and confidence intervals can be estimated using a *t*-distribution rather than a normal distribution to allow for the standard-error variability; several suggestions for the degrees of freedom (DF) have been published.^3,6^ The performance of these corrections is an area of active research in the context of longitudinal data,^13,14^ parallel CRTs,^15,16^ and SW-CRTs,^17,18^ and many of these papers have shown that GEE can be used with as few as 10 clusters using these corrections. However, research gaps remain for SW-CRTs, such as performance of the corrections with a binary outcome and varying cluster size.

In this paper, we begin by reviewing the literature studying several of the corrections suitable for SW-CRTs. We report the results of an extensive simulation study comparing the performance of these standard-error corrections and several DF estimators in the context of SW-CRTs with a binary outcome. We also assess whether GEE are robust to misspecification of the working correlation structure in this context. We provide guidance on which are suitable methods in this setting and demonstrate use of the methods under study in an illustrative example.

## 2. Generalised estimating equations with small-sample correction

There have been several corrections published to improve the performance of GEE with sandwich standard errors in small samples. In this paper we consider the following standard-error corrections and the use of a *t*-distribution. Details of these methods are given in Supplementary text 1.

### 2.1 Sandwich standard-error corrections

We consider several modifications that aim to reduce bias in the sandwich standard errors:

- Kauermann and Carroll (KC)^4^
- Fay and Graubard (FG)^3^
- Mancl and DeRouen (MD)^5^
- Morel, Bokossa, and Neerchal (MBN)^7^
- Mackinnon and White (MW)^2^

Some additional corrections were excluded because of computational complexity with varying cluster size that will limit their utility in CRTs.^6,14,19,20^

### 2.2 Degree-of-freedom corrections

In smaller samples, as well as correcting the standard-error estimator, *p*-values and confidence intervals may need to utilise the *t*-distribution to account for variability in the standard error estimation.

We consider the following estimators for *t*-distribution degrees of freedom (DF):

- Satterthwaite-type DF accounts explicitly for variability in the standard-error estimator. We consider estimators from Pan and Wall (DF_PW_) ^21^ and Fay and Graubard (DF_FG_).^3^
- Simpler estimators for the DF can be derived from the trial design. A common option is to use clusters minus parameters (DF_C-P_). Further trial design DF considered in this paper are described in section 4.

## 3. Literature review

The simplest correction, MW, has been found to give good standard error bias correction with a common cluster size in CRT and longitudinal data settings, leading to well-controlled type-one error when combined with DF_C-P_.^15,22^ However, it has not been studied in the SW-CRT setting. The performance is similar regardless of working correlation matrix.^13^

KC has been found to give standard errors with little bias in longitudinal data settings,^14^ CRTs,^23^ and SW-CRTS.^17^ Type-one error and confidence interval coverage have been well maintained with DF_C-P_, including variable cluster size with a coefficient of variation up to 0.6.^15^,^18^ Type-one error has often been inflated with the use of DF_Inf_^13,15,17,23^ and DF_PW,_^13^ but type-one error has sometimes been maintained with DF_Inf_ with common cluster size.^24^ Performance is similar regardless of working correlation matrix.^13,17^ We also identified variation in the expression^17^ and implementation^25–27^ of this correction, with approximations being used by some.^14,28^

Scott et al. show that FG is a simplification of KC.^17^ The performance of FG has been less clear. FG has been shown to give standard errors with low bias for SW-CRTs,^17^ but to overcorrect standard errors in a longitudinal setting with small cluster size.^14^ For SW-CRTs, type-one error and confidence interval coverage have been well maintained with DF_C-P_ and DF_FG_.^17,18^ For CRTs, type-one error and confidence interval coverage have been well maintained with DF_Inf_^24^ and DF_C-P_,^15^ but conservative with DF_FG_.^16^ For longitudinal data, DF_PW_ and DF_Inf_ have both resulted in inflated type-one error.^13^ Performance is similar regardless of working correlation matrix.^13,17^

MD has consistently been found to over correct the standard errors in longitudinal data,^19,29^ CRTs,^23,30^ and SW-CRTs.^17^ Generally, *p*-values and confidence intervals constructed with DF_Inf_^15,17,18,23,24,30^ and DF_PW_^13,14,30^ have had close to the correct size, although there have been exceptions to this.^13^ Conservative type-one error and confidence intervals have been observed with DF_C-P_.^15,18,30^ Performance may be improved with use of an independent working correlation matrix.^19^

MBN has been found to overcorrect standard errors for SW-CRTS.^17^ Type-one error remained inflated and confidence interval coverage remained low with DF_Inf_.^13,17,24,31^ Other DF have led to conservative type-one error in longitudinal settings.^13,14^ In the CRT settings, Li and Redden^15^ found that DF_C-P_ gave conservative type-one error with common cluster size, and inflated type-one error with varying cluster size. Performance is similar regardless of working correlation matrix.^13,17^

## 4. Simulation study methods

We conducted a simulation study to compare these small sample corrections. Simulation study parameters were selected to represent the most common characteristics of SW-CRTs, based on several recent reviews.^8,32,33^ Scenarios simulated are summarised in Table 1 and further details are given in Supplementary Text 2. Simulations were conducted in R v3.5.^34^

**Table 1.** Summary of data generation scenarios, see Supplementary Text 2 for more details.

Parameter	Phase one	Phase two
Control prevalence in the first measurement occasion	30%	30%
Time trend	Linear, odds ratio = 1.05 for each measurement occasion (6 occasions for all trial designs)	Linear, odds ratio = 1.05 for each measurement occasion (6 occasions for all trial designs)
Intervention effect	Odds ratio = 1.3	Odds ratio = 1.3
ICC within measurement occasion ( ρ )	0.01, 0.1	0.01, 0.05, 0.1
Correlation structures between measurement occasions	Exchangeable	Exchangeable
	AR(1) r = 0.6	AR(1) r = 0.6
	AR(1) r = 0.8	AR(1) r = 0.8
	AR(1) r = 0.8 with 50% less correlation between control and intervention	AR(1) r = 0.8 with 50% less correlation between control and intervention
Number of sequences	3,6	3,6
Number of clusters (C)	18	6,12,18, 24,
		42,48, 54
Cluster size	24, 60	24, 60,300^a^
Coefficient of variation (CV) of cluster size	0, 0.4	0, 0.4

^a^The scenario with cluster size of 300 was only run with exchangeable within cluster correlation, 12, 24, and 42 clusters, and no variability in cluster size.

All combinations of parameters were generated unless otherwise stated, and we simulated 1000 datasets for each scenario. This gave 95% probability of estimating confidence-interval coverage between 93.6% and 96.4% when there is true 95% coverage.

The simulations were conducted in two phases. Phase one included 64 scenarios with 18 clusters to identify the small-sample corrections that gave good coverage in the widest range of scenarios. Phase two involved a wider range of 690 scenarios with a smaller number of small sample corrections chosen based on the results of phase one.

### 4.1 Data generation process

#### 4.1.1 Method used to generate binary data

In order to simulate binary data specifying the marginal effects, we used the method of Emrich and Piedmonte.^35^ Observations are sampled from a multivariate-normal distribution with zero means. The covariance matrix for the multivariate-normal distribution is determined using an algorithm, so that the dichotomised data will have the desired correlation structure on the proportion scale. The sampled values are dichotomised at values that give the required marginal probabilities.

#### 4.1.2 Prevalence and intervention effects

We simulated a repeated cross-sectional design with data collected at six measurement occasions. Individuals were each observed once and different individuals were observed in each measurement occasion. We simulated an outcome prevalence of 30% (odds = 0.43) in the control condition in the first measurement occasion. This increased with a linear (on the log scale) trend with OR = 1.05 at each measurement occasion, so that by the sixth occasion, the outcome prevalence in the control condition would have been 37% (odds = 0.43 × 1.05^5^ = 0.55). This did not change with the trial design to facilitate comparability of the underlying data. The intervention effect had OR = 1.3. These characteristics were the same in both phases of the simulations.

#### 4.1.3 Correlation structures

In phase one of the simulations, we simulated data with either a high (0.1), or low (0.01) intracluster correlation coefficient (ICC) within each measurement occasion on the proportion scale ( ρ in Supplementary Text 2). For phase two, we introduced an additional medium (0.05) ICC.

The correlation structures were the same in both phases of the simulations. Within each cluster and measurement occasion, observations were exchangeable on the proportions scale. We simulated four within-cluster correlation structures to vary the correlation between the six measurement occasions:

- Exchangeable: All observations within a cluster were equally correlated to one another.
- Autoregressive (AR(1)) with autoregressive parameter r_0_=0.6: The correlation between observations in the same cluster but different measurement occasions reduced by 40% with each successive occasion.
- AR(1) r_0_=0.8: The correlation between observations in the same cluster but different measurement occasions reduced by 20% with each successive occasion.
- AR(1) r_0_=0.8 + 50% reduction in correlation between control and intervention observations: The correlation between observations in the same cluster reduced by 20% with each successive measurement occasion, and there is an additional 50% reduction in correlation between observations where one is in the control condition and the other the intervention condition. This could occur if the intervention effect varied between clusters.

#### 4.1.4 Trial designs

In both phases, we simulated data from SW-CRT designs with either three or six sequences, as shown in Figure 1. In both designs, there was no period with all clusters in the control as inclusion of this period is inefficient.^36,37^ In the design with six sequences, the trial periods coincided with the measurement occasions. In the design with three sequences, each trial period contained two measurement occasions.

In phase one, we simulated trials with 18 clusters. In phase two, we simulated trials with 6, 12, 18, 24, 42, 48, and 54 clusters; this allowed us to explore the performance of the corrections with a range of small trials and explore the behaviour of uncorrected GEE for larger trials.

In both phases, we simulated trials with common and varying (coefficient of variation (CV) = 0.4) cluster size. Varying cluster size was sampled from a negative binomial distribution with the required mean and standard deviation so that each measurement occasion had a minimum of two observations for each cluster. In phase one, we simulated clusters with 24 or 60 observations per cluster; where clusters varied in size, this was the mean. A cluster size of 24 corresponds to four observations in each measurement occasion, and a cluster size of 60 corresponds to 10 observations in each measurement occasion. In phase two, we introduced a limited number of scenarios with 300 observations per cluster (50 observations per measurement occasion). To maintain a feasible computational time, this scenario was only introduced with common cluster size, exchangeable correlations, and 12, 24, or 42 clusters.

#### 4.1.5 Analysis methods

All datasets were analysed at the individual level using GEE with sandwich standard errors with both an independent and exchangeable working correlation matrix using the R package geepack ^38^. The GEE used a binomial distribution with logistic link. The models included an intervention effect and a period effect, with a category for each trial period.

In phase one, all corrected sandwich estimators described in section 2.1 were calculated using the R package geesmv ^25^ with default values for boundary and adjustment values where relevant. For the two standard error corrections that consistently gave the least biased standard errors, confidence intervals were created using a normal distribution and t-distribution with the following DF:

- Pan and Wall (DF_PW_)^21^: calculated by the R package geesmv V1.3.
- Fay and Graubard (DF_FG_)^3^: calculated by the R package saws V0.9-6.1.
- Clusters minus parameters (DF_C-P_): With the trial designs we used, the number of parameters is *number of sequences + 1*. In scenarios with one cluster randomised to each sequence, this will give a negative result, which we replaced with 1 DF.
- Cluster periods minus parameters (DF_CP-P_): Uses cluster periods as the observation unit, and parameters are as above.
- Cluster periods minus parameters with clusters regarded as additional parameters (DF_CP-C-P_): This is based on an ANOVA-style decomposition with clusters as parameters.

The two selected standard errors and two DF that resulted in confidence interval coverage nearest to the 95% level were carried forward from phase one to phase two.

##### 4.1.6 Post-hoc analysis method

Our simulation study identified problems with the stability of the KC standard error from the geesmv package. To address this problem, we repeated the simulation using an approximation to the KC correction (KC-approx) suggested by Gallis et al.^28^ and implemented in xtgeebcv in Stata. More details are given in Supplementary text 1.

### 4.2 Analysis of simulation results

We calculated convergence proportions, standardised bias of intervention effect estimates, and the relative error of standard errors *([mean estimated standard error]/[standard deviation of the intervention effect estimates across simulations] − 1*). We calculated the mean and standard deviation of DF_PW_ and DF_FG_; other DF were determined by the trial design.

We refer to 95% confidence interval coverage (the proportion of results with 95% confidence intervals that contained the true effect) below 93.6% as low coverage or under covered, and coverage above 96.4% as high coverage or over covered. Power was calculated as the proportion of datasets with *p* < 0.05 against the null hypothesis of no intervention effect.

We used linear regression models to quantify the association between simulation scenario characteristics and analysis method performance measures such as bias and coverage.

## 5. Simulation study results

### 5.1 Phase one

Few analyses failed to converge (<4% in any scenario), and the intervention effect estimate bias was small in all scenarios (≤10% standardised bias in any scenario, Supplementary Table 1 and Supplementary Figure 1).

#### 5.1.1 Standard error bias

**Figure 2.**
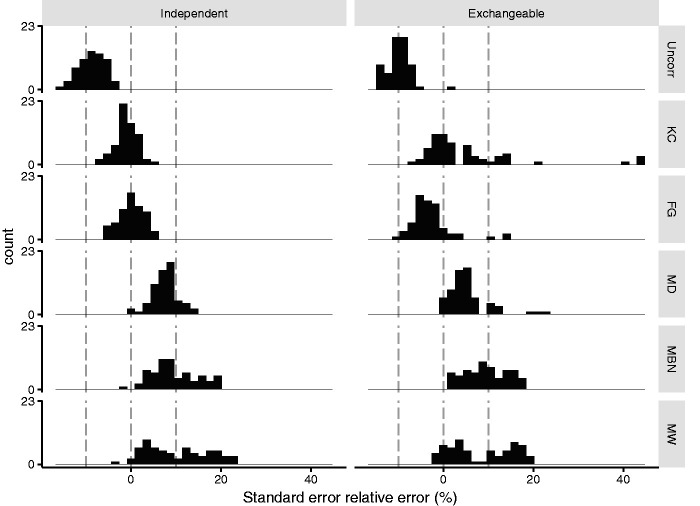
Phase one: Histogram of relative error of sandwich standard errors with each small sample correction (rows: Uncorrected (Uncorr), Kauermann and Carroll (KC), Fay and Graubard (FG), Mancl and DeRouen (MD), Morel, Bokossa, and Neerchal (MBN), and Mackinnon and White (MW)) for an independent and exchangeable working correlation matrix (columns). All scenarios have 18 clusters. Grey dotted lines indicate 0% error and ±10% error.

Figure 2 shows histograms of the relative error of standard errors in each scenario. Uncorrected standard errors were underestimated by an average of 9% and 10% with a working independent and exchangeable correlation matrix, respectively. All corrections gave standard errors closer to the empirical standard deviation. Overall, KC and FG gave the most accurate standard errors and were the two corrections carried forward in the simulations. Below, we provide details of the performance of each correction and Supplementary Table 2 shows regression analyses for each correction.

*Kauermann and Carroll:* With an independent working correlation matrix, KC standard errors were always within 10% of the empirical standard deviation. With an exchangeable working correlation matrix, KC were on average unbiased, but became more variable: KC overcorrected standard errors by >10% in 13/64 (20%) scenarios: this occurred mostly (11/13) with exchangeable or AR(1) r = 0.8 correlations, so that the correlation structure was correctly or close to correctly specified, (Supplementary Figure 2) and with ICC = 0.1 (Supplementary Figure 3). Bias was similar for scenarios with common and varying cluster size (Supplementary Table 2).

*Fay and Graubard:* With an independent working correlation matrix, the FG standard errors were within 10% of the empirical standard deviation in all scenarios. With an exchangeable working correlation matrix, standard errors were undercorrected with ICC = 0.1 but close to empirical values with ICC = 0.01 (mean=−1% ICC = 0.01 vs. −5% ICC = 0.1, Supplementary Figure 3).

*Mancl and DeRouen:* MD standard errors overcorrected by on average 6%. Over correction was greater when cluster size varied (8% CV = 0.4 vs. 5% CV = 0, Supplementary Figure 4) and with low ICC (8% ICC = 0.01 vs. 5% ICC = 0.1, Supplementary Figure 3).

*Morel, Bokossa, and Neerchal:* MBN standard errors overcorrected by on average 6% with three sequences and 13% with six sequences (Supplementary Figure 5). Bias was on average 3% larger for common cluster size than varying cluster size (Supplementary Table 2).

*Mackinnon and White:* MW standard errors followed a similar trend to MBN standard errors, over correcting on average 3% with three sequences and 16% with six sequences (Supplementary Figure 5). Bias was on average 4% larger for common cluster size than varying cluster size (Supplementary Table 2).

#### 5.1.2 Degrees of freedom

Table 2 summarises estimated DF with FG standard errors and an independent working correlation matrix. Results were similar with different analysis methods. Supplementary Table 3 shows factors associated with larger DF.

**Table 2.** Phase one: The mean of the within scenario mean and standard deviation (SD) of all degrees of freedom (DF) by number of sequences.

Sequences	DF_C-P_	DF_CP-P_	DF_CP-C-P_	DF_FG_ mean (SD)	DF_PW_ mean (SD)
3	14	50	33	13 (1.2)	69 (70.3)
6	11	101	84	12 (1.2)	53 (52)

Note: All calculated with Fay and Graubard standard errors and an independent working correlation matrix.

DF_FG_ were of similar magnitude to DF_C-P_. There was little variability within scenarios (mean standard deviation (SD)=1.2).

DF_PW_ were larger than DF_FG_ and had large variability within scenarios (mean SD = 61.8).

#### 5.1.3 Coverage of 95% confidence intervals

**Figure 3.**
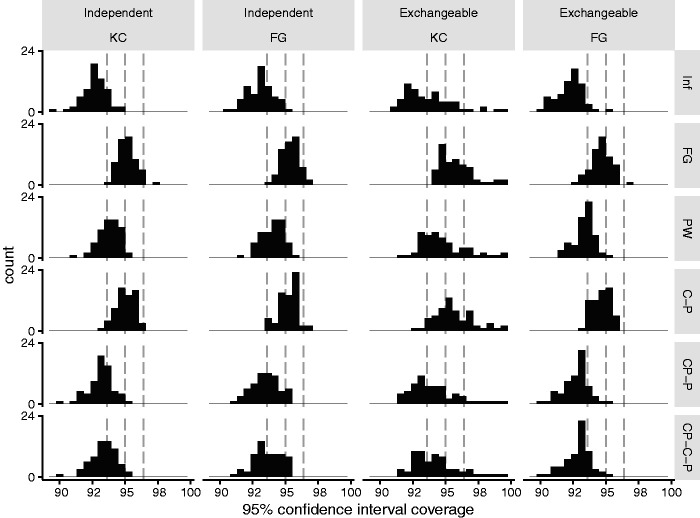
Phase one: Histogram of 95% confidence interval coverage of Kauermann and Carroll (KC) and Fay and Graubard (FG) standard errors and each working correlation matrix (columns) with each *t*-distribution degree of freedom estimator (rows, infinite (Inf, a normal distribution), Fay and Graubard (FG), Pan and Wall (PW), clusters minus parameters (C-P), Cluster periods minus parameters (CP-P), Cluster periods minus clusters minus parameters (CP-C-P)). Grey dotted lines indicate 93.6%, 95%, and 96.4% coverage.

Figure 3 shows histograms of 95% confidence interval coverage for KC and FG standard errors by working correlation matrix and DF. With both FG and KC standard errors, coverage was consistently closer to 95% using a *t*-distribution with either DF_C-P_ or DF_FG_ than other DF (Supplementary Table 4). These four analysis methods were continued forward to phase two. Other DF resulted in low coverage.

With an independent working correlation matrix, FG and KC standard errors gave consistent coverage with both DF_C-P_ and DF_FG_. With an exchangeable working correlation matrix, coverage largely followed the patterns of bias in standard errors. KC standard errors had variable coverage due to over correction of standard errors in some scenarios: this was unaffected by choice of DF. FG standard errors had good coverage with low ICC and either DF (mean coverage 95.1% and 94.8% with DF_FG_ and DF_C-P_ respectively), but a small degree of under coverage with high ICC and either DF due to undercorrection of standard errors (94.3% and 94.5% with DF_FG_ and DF_C-P_, respectively).

### 5.2 Phase two

In phase two, we widened the range of scenarios to include between 6 and 54 clusters, and a larger cluster size of 300, and only considered KC and FG standard errors with DF_C-P_ and DF_FG_. Similar to phase one, non-convergence was uncommon: <5% failed to converge in any scenario. There was no indication of intervention effect estimate bias in any scenario (≤11% standardised bias, Supplementary Table 5 and Supplementary Figure 6).

#### 5.2.1 Standard error bias

**Figure 4.**
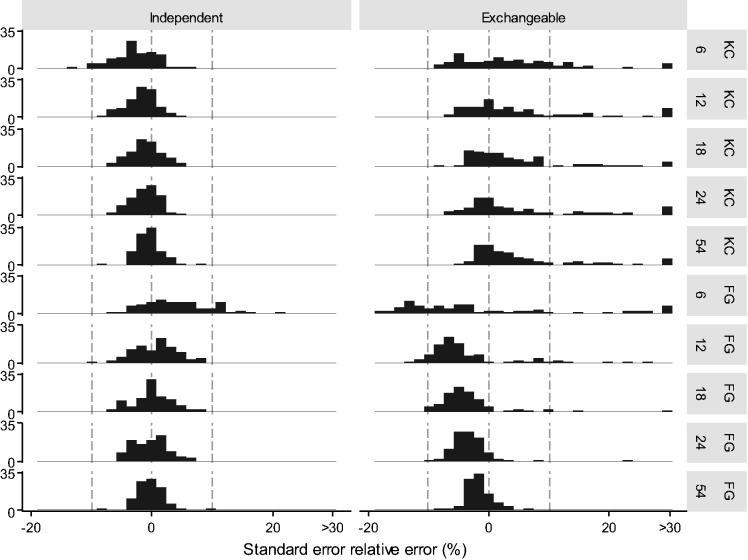
Phase two: Histogram of relative error of sandwich standard errors with an independent or exchangeable working correlation matrix (columns) for Kauermann and Carroll (KC), and Fay and Graubard (FG) standard errors with either 6, 12, 18, 24, or 54 clusters (rows). Grey dotted lines indicate 0% error and ±10% error.

Figure 4 shows relative error of KC and FG standard errors (also see Supplementary Table 6).

*Kauermann and Carroll:* With an independent working correlation matrix, KC standard errors remained unbiased with as few as six clusters. There was a small increase in the variability of estimates with fewer clusters. With an exchangeable working correlation matrix, the variability of KC standard errors increased with fewer clusters (Figure 4). The over-correction with an exchangeable working correlation matrix and a true exchangeable correlation structure became more severe with a cluster size of 300 (mean 91% relative error).

*Fay and Graubard:* With an independent working correlation matrix, FG standard errors had minimal bias with 12 clusters, but were on average 4% larger than the standard deviation of the intervention effect estimates with six clusters. With an exchangeable working correlation matrix, they became more variable with fewer clusters. The under correction with ICC = 0.1 became more severe with fewer clusters (mean = −7% with 12 clusters and −12% with 6 clusters), and overcorrection was seen with ICC = 0.01 (mean = 2% with 12 clusters and 13% with 6 clusters, Supplementary Figures 7).

*Uncorrected:* Bias of uncorrected sandwich standard errors increased with fewer clusters, but some remained with as many as 54 clusters: they were on average 14% smaller than the standard deviation of the intervention effect estimates with 12 clusters and 3% smaller with 54 clusters (Supplementary Figure 8).

#### 5.2.2 Degrees of freedom

With six clusters, DF_FG_ was on average two DF larger than DF_C-P_. As the number of clusters increased, the difference reversed direction (Supplementary Figure 9): with 42 clusters, DF_FG_ was on average four DF smaller than DF_C-P_.

#### 5.2.3 Coverage of 95% confidence intervals

**Figure 5.**
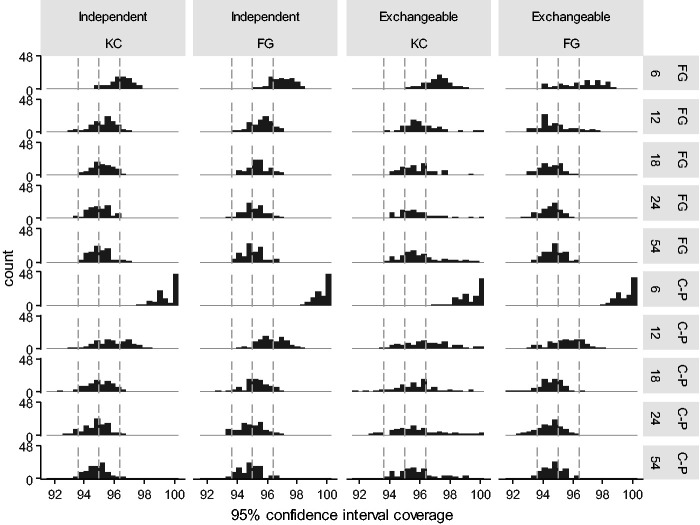
Phase two: Histogram of 95% confidence interval coverage of Fay and Graubard (FG), and Kauermann and Carroll (KC) standard errors and each working correlation matrix (columns) with 6, 12, 24, or 48 clusters using DF_FG_ and DF_C-P_ (rows). Grey dotted lines indicate 93.6%, 95%, and 96.4% coverage.

Coverage was consistently close to 95% with DF_FG_, an independent working correlation matrix, and either standard error (Figure 5 and Supplementary Table 7). DF_C-P_ also performed well with 18 or more clusters with six sequences, and 12 or more clusters with three sequences.

With an exchangeable working correlation matrix, KC standard errors gave variable but conservative coverage: Only 9/690 (1%) scenarios had low coverage with DF_FG_. With FG standard errors, under correction of standard errors consistently resulted in coverage slightly less than 95%: 54/690 (8%) scenarios had low coverage with DF_FG_.

The uncorrected GEE gave confidence intervals with low coverage in all scenarios with 24 or fewer clusters (Supplementary Figure 10). With 54 clusters, confidence intervals based on a normal distribution had low coverage in 84/192(44%) scenarios and coverage remained low using DF_C-P_ (32/192[26%]) or DF_FG_ (21/192[11%]).

#### 5.2.4 Power

With an independent working correlation matrix, KC and FG standard errors led to similar power (median 0.5% more power with KC IQR 0% to 0.9%, Supplementary Figure 11). DF_C-P_ led to median 4% lower power than DF_FG_ with six clusters, 1% lower with 12 clusters, and similar power with 18 or more clusters (Supplementary Figure 12).

With KC standard errors and DF_FG_, an exchangeable working correlation matrix led to similar power to an independent working correlation matrix (median 0.4% more power with an independent working correlation matrix, IQR -1.3% to 0.4%, Supplementary Figure 13).

With FG standard errors and DF_FG_, an exchangeable working correlation matrix led to an average 1.8% higher power than an independent working correlation matrix with 24 observations per cluster, 5.2% higher with 60 observations per cluster, but 39.6% higher power with 300 observations per cluster (Supplementary Figure 13).

### 5.3 Post-hoc KC approximation

With an independent working correlation matrix, KC-approx was similar to KC and showed minimal bias with as few as six clusters (Supplementary Figure 14). This lead to confidence intervals with close to 95% coverage with 12 or more clusters and over coverage with six clusters with either DF_FG_ or DF_CMP_.

With an exchangeable working correlation matrix, KC-approx standard errors had similar performance to FG standard errors, and did not suffer from the overcorrection seen with KC standard errors (Supplementary Figure 14). Standard errors showed little bias with 42 or more clusters, but were undercorrected with 24 or fewer clusters, with the degree of undercorrection increasing with fewer clusters (mean under correction = 2% with 24 clusters and 10% with 6 clusters). Despite this, confidence interval coverage was close to nominal levels when the correlation structure was correctly specified (4/186 [2%] scenarios had under covered confidence intervals with DF_FG_). However, confidence interval coverage was slightly low in scenarios with reduced correlation between control and intervention observations (13/168 [8%] scenario with low coverage, Supplementary Figures 15 and 16).

Power followed similar patterns to FG with small increase in power using an exchangeable working correlation structure with 24 and 60 observations per cluster (mean 1.5% and 4.0% higher), but larger differences with 300 observations per cluster (mean 37.0% higher).

## 6. Illustrative example

To explore the impact of choice of analysis, we will look at an example trial analysed with each method investigated in this study. Trajman et al.^39^ conducted a trial comparing a new TB diagnostic test Xpert MTB/RIF to the commonly used smear microscopy test. We will focus on the impact of the type of test on the proportion of cases with a laboratory confirmation of diagnosis, which was a secondary outcome of the trial. This is an example of a continuous recruitment design with individuals sampled throughout the trial and one outcome collected from each.^40^ The trial randomised 14 laboratories (clusters) into seven sequences. In the first month of the trial, all laboratories were using smear microscopy. Then each month, two laboratories switched to using the new tests, so that all laboratories were using the new diagnostic test in the eighth month of the trial. There were a total of 3924 observations, with a mean of 136 observations per cluster and CV in cluster size of 0.48.

Table 3 shows the results of this trial analysed with all GEE analyses considered in this simulation study. Each model included a categorical variable for month to adjust for time and a variable for intervention status.

**Table 3:** Example TB diagnostic trial analysed with each GEE method.

	Working correlation matrix	
	Independent	Exchangeable
Odds ratio estimate	1.65	1.51
Standard error (percentage increase compared to uncorrected standard error)^a^		
Uncorrected	0.1684	0.1115
FG	0.2056 (+22%)	0.1196 (+7%)
KC	0.1891 (+12%)	0.1948 (+75%)
MD	0.2198 (+30%)	0.1395 (+25%)
MBN	0.1956 (+16%)	0.1422 (+28%)
MW	0.2818 (+67%)	0.1865 (+67%)
KC approximation	0.1921 (+14%)	0.1245 (+12%)
Degrees of Freedom		
Pan and Wall^b^	70.1	17.9
Fay and Graubard^b^	7.6	10.5
Clusters minus parameters	5	5
Cluster periods minus parameters	103	103
Cluster periods minus parameters with clusters regarded as parameters	89	89
(95% CI) *p*-value		
Uncorrected	(1.19, 2.30) *p* = 0.003	(1.22, 1.88) *p* < 0.001
FG + DF_FG_	(1.02, 2.66) *p* = 0.04	(1.16, 1.97) *p* = 0.006
FG + DF_C-P_	(0.97, 2.80) *p* = 0.06	(1.11, 2.06) *p* = 0.02
KC approx. + DF_FG_	(1.06, 2.58) *p* = 0.03	(1.15, 1.99) *p* = 0.007
KC approx. + DF_C-P_	(1.01, 2.71) *p* = 0.05	(1.10, 2.08) *p* = 0.02

FG: Fay and Graubard; KC: Kauermann and Carroll; MD: Mancl and DeRouen; MBN: Morel, Bokossa, and Neerchal; MW: Mackinnon and White; GEE: Generalised estimating equations.

^a^Standard errors given on the log odds ratio scale.

^b^Using Fay and Graubard standard error.

Standard error inflation by the small sample corrections reflected the results of the simulation study. FG standard errors with an exchangeable working correlation matrix gave the smallest correction (7% inflation of the uncorrected standard errors). The largest correction was by the KC standard errors with an exchangeable working correlation matrix (75% inflation of the uncorrected standard errors) demonstrating the overcorrection of standard errors observed in the simulation study. The KC approximation gave a smaller inflation (14% increase from the uncorrected standard errors). MW standard errors were also large compared to the other corrected standard errors.

The most conservative DF are given DF_C-P_ (DF_C-P_=5). Consistent with the simulation study, DF_FG_ were slightly larger (DF_FG_=7.6 with an independent and 10.5 with an exchangeable working correlation matrix). All other DFs were larger; most were over 70.

Table 3 also shows confidence intervals and *p*-values with the methods found to work consistently well by the simulation study (FG and KC-approx standard errors, with DF_FG_ and DF_C-P_) and with no small-sample correction. The uncorrected analyses conclude that there is strong evidence that the new test increased the odds of a confirmed diagnosis (*p* = 0.003 and *p* < 0.001 with an independent and exchangeable working correlation matrix, respectively). With an independent working correlation matrix, all corrected methods lead to similar conclusions (0.03 ≤ *p* ≤ 0.06) with confidence intervals with a similar width. With an exchangeable working correlation matrix, all methods led to some evidence of an effect, DF_FG_ leading to greater evidence (*p* = 0.02 with DF_C-P_ and *p* = 0.007 for FG and *p* = 0.009 for KC-approx with DF_FG_) .

## 7. Discussion

We found that, with as many as 54 clusters, uncorrected sandwich standard errors were smaller than the standard deviation of the intervention effect estimates across simulations leading to lower than nominal 95% confidence interval coverage. KC and FG standard errors performed well to mitigate this bias when used with an independent working correlation matrix, and results were robust to misspecification of the working correlation matrix. Use of a *t*-distribution with DF_FG_ gave consistently good confidence interval coverage with at least 12 clusters, and DF_C-P_ gave similar results with at least 18 clusters. Both DF became conservative with fewer clusters. Interpretation of results with an exchangeable working correlation matrix is less clear. KC standard errors required use of an approximation. A small amount of bias remained with FG standard errors leading to coverage less than 95%. An exchangeable working correlation matrix gave higher power than an independent working correlation matrix with 300 observations per cluster, but similar power with smaller clusters.

We found that KC standard errors were unbiased when used with an independent working correlation matrix with cluster size from 24 to 300, CV of cluster size 0 to 0.4, ICC from 0.01 to 0.1, across different true correlation structures, with 3 or 6 sequences, and with as few as 12 clusters. These findings are consistent with previous research that reported good overall performance.^13,16–18,24^ With an exchangeable working correlation matrix, we found that KC were highly variable in some scenarios due to instability in the matrix inversion required by this correction.^14,28^ The approximation we tested, also used in xtgeebcv in Stata,^28^ corrected this instability, with a small cost to bias correction: similar to the FG standard errors, a small bias remained with 24 clusters or fewer. Other CRT and SW-CRT simulations have not encountered the problem of instability with similar cluster size and ICC without describing the use of alternative calculations,^18,23^ although this could be due to differences in implementation that have not been reported. Our results highlight the importance of the implementation of this correction. Other solutions to the complex inversion such as singular value decomposition (used by the SAS glimmix procedure ^26^) may have better performance than the methods we explored.

We found that FG standard errors were unbiased with an independent working correlation matrix with cluster size from 24 to 300, CV of cluster size from 0 to 0.4, ICC from 0.01 to 0.1, across different true correlation structures, with three or six sequences, and with as few as 12 clusters. We observed a small bias in standard errors when used with an exchangeable working correlation matrix leading to a small degree of under coverage of confidence intervals. Previous studies have concluded that FG standard errors perform well in similar scenarios.^13,16–18,24^ The small size of this bias and impact on confidence interval coverage is likely why the under correction has not previously been reported. Consistent with our findings, Scott et al. and Leyrat et al. observed conservative confidence interval coverage and type-one error, respectively, with FG and DF_FG_ and a very small number of clusters^16,17^ in SW-CRTs and CRT, respectively.

Our finding that KC and FG provide more consistent standard error estimates with an independent working correlation matrix is novel in the literature. We also found that this had limited impact on power for KC with any cluster size, and FG with median to small cluster size, but a large impact on power for FG with large clusters. The KC findings are contrary to the literature on GEE with sandwich standard errors that suggest power will be higher the closer the working correlation structure is to the true correlation structure.^41^ These comparisons of power should be viewed with some caution, as some of the difference will be mediated by the bias in standard errors with an exchangeable working correlation matrix. The drawback to assuming independence is that a separate analysis is required to estimate the correlation parameters, which CONSORT guidelines recommend reporting.^42^

Overcorrection of MD, MW, and MBN standard errors has also been reported elsewhere.^15,17,23,24^ Where others have found appropriate type-one error and confidence interval coverage using MD, this may be explained by the choice of DF_Inf_^15,17,18,23,24,30^ and DF_PW_.^13^,^14,30^ Our findings do not support the use of an average of KC and MD standard errors as suggested by others^14^: our results imply that this would result in standard errors that are too large. For MBN and MW standard errors, contrary to findings for parallel CRTs,^15,22,24^ we observed overcorrection with common cluster size as well as varying cluster size, and moreover, the bias was larger with common cluster size.

We have also given a detailed comparison of the performance of DF_FG_ and DF_PW_ in comparison to simpler estimators based on the trial design.

DF_PW_ performed poorly: we found large variability in the estimates, a property that has not been examined in other simulation studies that have included this method.^6,43^

DF_FG_ gave less variable estimates than DF_PW_ and provided the most robust confidence interval coverage of the DF we considered. Others have also found that this method performs well.^3,17^ We found that DF_FG_ were similar to DF_C-P_ with 18 or more clusters and so lead to similar coverage. With fewer than 18 clusters, DF_FG_ was larger than DF_C-P_ and had closer to 95% coverage. Other than by number of clusters, the difference in performance varied little between the scenarios we considered including common and varying cluster size.

### 7.1 Strengths and limitations

The strengths of this study are in the comprehensive range of scenarios considered (nearly 700 in phase two), the wide range of small sample corrections included, and the use of methods that generate correlated data from marginal effects.

There are several corrections that we did not consider, which may have superior performance to the corrections we used.^6,14,19,20^ However, software availability for varying cluster size currently limits the utility of these additional corrections for CRTs. Our approach has been to identify methods that performed well across a broad range of scenarios; an alternative correction may have better performance in a particular scenario than those we have concluded were robust overall. We only considered a common outcome, and we have focused on a binary outcome, where GEE estimate marginal, or population average, effects: researchers should consider whether this is the effect of interest in their setting.^44^ Our simulation study used an SW-CRT design with no baseline period. However, our results are consistent with other simulation studies that included a baseline period.^17,18^ The working correlation structures we considered (exchangeable and independent) did not incorporate the timing of observations within clusters. We have shown that this did not affect the confidence interval coverage, but as is typically the case with GEE, power may be improved by choosing a working correlation matrix that more closely follows the true structure. For example, the working correlation matrix could allow correlation to decay with distance between observation periods.^45^

### 7.2 Concluding remarks

We have shown that GEE with small sample corrections are a robust method of analysis in a range of settings where SW-CRTs are commonly used. When there are fewer than 50 clusters, we recommend the use of KC, KC-approx, or FG standard errors with an independent working correlation structure, KC-approx standard errors with an exchangeable working correlation structure if clusters are large, or FG standard errors with an exchangeable working correlation structure if clusters are large and ICC is low. A *t*-distribution is also required with DF_FG_, although DF_C-P_ is sufficient for trials with 18 or more clusters.

## Supplemental Material

sj-pdf-1-smm-10.1177_0962280220958735 - Supplemental material for Comparison of small-sample standard-error corrections for generalised estimating equations in stepped wedge cluster randomised trials with a binary outcome: A simulation study Supplemental material, sj-pdf-1-smm-10.1177_0962280220958735 for Comparison of small-sample standard-error corrections for generalised estimating equations in stepped wedge cluster randomised trials with a binary outcome: A simulation study by JA Thompson, K Hemming, A Forbes, K Fielding and R Hayes in Statistical Methods in Medical Research
