# Supplementary material for: Comparison of small-sample standard-error corrections for generalised estimating equations in stepped wedge cluster randomised trials with a binary outcome: A simulation study
Source: Stat Methods Med Res. 2020 Sep 24;30(2):425–39. doi: 10.1177/0962280220958735 (PMC8008420; doi:10.1177/0962280220958735)

Supplementary Material

Supplementary text 1: Generalised Estimating Equation methods

Denote Y_{il} as the binary outcome in cluster $i = 1, ..., C$ for observations $l = 1, ..., m_i$, and Z_{il} as a p-dimensional covariate vector for p covariates. The marginal mean $E[Y_{il}|Z_{il}] = \mu_{il}$ where $g(\mu_{il}) = \beta Z_{il}$ for some link function $g(.)$ and p-vector of coefficients β.

The GEE estimates $\hat{\beta}$ are found by solving the estimating equations

$$\sum_{i=1}^{C} D_i^T V_{Wi}^{-1}(Y_i - \mu_i) = 0$$

where $D_i = \partial\mu_i/\partial\beta'$, V_{Wi} is the covariance matrix of Y_i assuming working correlation matrix R_W. Estimates of $\hat{\beta}$ will be asyptotically consistent regardless of specification of R_W [1].

Uncorrected sandwich variances are calculated as follows:

$$V_S = V_M \left[\sum_{i=1}^{C} D_i^T V_{Wi}^{-1} Cov(Y_i) V_{Wi}^{-1} D_i \right] V_M$$

where $V_M = \left(\sum_{i=1}^{C} D_i^T V_{Wi} D_i \right)^{-1}$ is the model based variance, and $Cov(Y_i) = (Y_i - \hat{\mu}_i)(Y_i - \hat{\mu}_i)^T$ is an estimator of the covariance matrix of Y_i. When V_{Wi} is correctly specified, $V_S = V_M$.

Sandwich variance corrections

The sandwich variance estimator is biased with a small number of clusters. The modificatied estimators below aim to reduce this bias.

Kauermann and Carroll (KC) [2] derived the following variance estimator:

$$V_{KC} = V_M \left[\sum_{i=1}^{C} D_i^T V_{Wi}^{-1} A_{KCi} Cov(Y_i) A_{KCi} V_{Wi}^{-1} D_i \right] V_M$$

where $A_{KCi} = (I_i - H_i)^{-1/2}$, I_i is an $m_i \times m_i$ identity matrix and $H_i = D_i V_M D_i^T V_{Wi}^{-1}$ is an expression for the leverage of cluster i

The calculation of $A_{KCi} = (I_i - H_i)^{-1/2}$ using eigenvalue decomposition (as implemented in geesmv [12]) can lead to erroneous results[3]. Several alternative methods for calculation of KC have been suggested. We implement a **Kauermann and Carroll approximation (KC-approx)** described by Gallis et al [4]:

$$V_{KC-approx} = V_M \frac{\left[\sum_{i=1}^{C} D_i^T V_{Wi}^{-1}(I_i - H_i)^{-1} Cov(Y_i) V_{Wi}^{-1} D_i + \sum_{i=1}^{C} D_i^T V_{Wi}^{-1} Cov(Y_i)(I_i - H_i^T)^{-1} V_{Wi}^{-1} D_i \right]}{2} V_M$$

Fay and Graubard (FG) [5] derived the variance estimator:

$$V_{FG} = V_M \left[\sum_{i=1}^{C} A_{FGi} D_i^T V_{Wi}^{-1} Cov(Y_i) V_{Wi}^{-1} D_i A_{FGi} \right] V_M$$

where $A_{FGi} = diag\left(\left[1 - min\left(b, [D_i^T V_{Wi} D_i V_M]_{jj}\right)\right]^{-1/2}\right)$. We set b=0.75 [5].

Mancl and DeRouen (MD) [6] derived the variance estimator:

$$V_{MD} = V_M \left[\sum_{i=1}^{C} D_i^T V_{Wi}^{-1} A_{MDi} Cov(Y_i) A_{MDi} V_{Wi}^{-1} D_i \right] V_M$$

where $A_{MDi} = (I_i - H_i)^{-1}$.

Morel, Bokossa and Neerchal (MBN) [7] derived the variance estimator:

$$V_{MBN} = V_M \left[\sum_{i=1}^{C} D_i^T V_{Wi}^{-1} A_{MBNi} V_{Wi}^{-1} D_i \right] V_M$$

where

$$A_{MBNi} = kCov(Y_i) + \delta \phi V_{Wi}$$

$$k = \frac{\sum_{i=1}^{C} m_i - 1}{\sum_{i=1}^{C} m_i - p} \frac{C}{C-1}$$

$$\delta = max\left(\frac{p}{C-p}, \frac{1}{d}\right)$$

$$\phi = max\left(f, trace\left[V_M \sum_{i=1}^{C} D_i^T V_{Wi}^{-1} Cov(Y_i) V_{Wi}^{-1} D_i^T\right] /p\right)$$

We set $d = 2$ and $f = 1$.

Mackinnon and White (MW) [8] suggest the simple estimator

$$V_{MK} = \frac{C-p}{p} V_S$$

Degree of freedom corrections

Satterthwaite-type

Pan and Wall ($\mathbf{DF_{PW}}$) [9] derive the estimator

$$DF_{PW} = \frac{2E[V_R]^2}{Var[V_R]}$$

where V_R is a corrected sandwich variance as described above, and $Var[V_R]$ can be extracted from

$$Cov(Vec[V_S]) = C^2 (V_M \otimes V_M) \left(\sum_{i=1}^{C} \frac{[P_i - \bar{P}][P_i - \bar{P}]^T}{C(C-1)} \right) (V_M \otimes V_M)$$

where $P_i = vec[D_i^T V_{Wi}^{-1} Cov(Y_i) V_{Wi}^{-1} D_i^T]$

Fay and Graubard ($\mathbf{DF_{FG}}$)[5] derive the estimator

$$DF_{FG} = \frac{trace(\tilde{\psi}B_1)^2}{trace(\tilde{\psi}B_1\tilde{\psi}B_1)}$$

where $\tilde{\psi} = diag[\tilde{\psi}_1, ..., \tilde{\psi}_C]$

$$\tilde{\psi}_i = w_i \left(\sum_{l=1}^{C} w_l\right) \left(\sum_{l=1}^{C} A_{FGl} D_l^T V_{Wl}^{-1} Cov(Y_l) V_{Wl}^{-1} D_l^T A_{FGl}\right)$$

$w_i = K^T \left(\left[\sum_{j\neq i} D_j^T V_{Wj} D_j\right]^{-1} - V_M\right) K$, K is a vector indicating the null hypothesis $K^T\beta = K^T\beta_0$,
$B_1 = G^T M G$,
$G = I_{PK} - diag[D_1^T V_{W1} D_1, ..., D_C^T V_{WC} D_C] V_M [I_P, ..., I_P]^T$, and
$M = diag[A_{FG1} V_M K K^T V_M A_{AG1}, ..., A_{FGC} V_M K K^T V_M A_{AGC}]$.

Supplementary text 2: Simulation study methods

Data genaration

Generating varied cluster size

Scenarios with varying cluster size had a cluster size sampled from a negative-binomial distribution to give minimum 12 observations per cluster (minumim 2 observations in each measurement occasion) and a coefficient of variation of 0.4.

The coefficient of variation is defined as $CV = S/m$ where S is the standard deviation of the cluster size, and m is the mean cluster size.

If we sample each measurement occasion within each cluster instead, the mean size of each cluster measurement occasion $n = m/6$ and variance of each cluster measurement occasion $S_n^2 = S^2/6$

Shifting the distribution so that the minimum sample in each cluster period is 2, the number of observations in each cluster measurement occasions are generated as $n_{ij} = 2 + \delta_{ij}$ where

$$\delta_{ij} = NB\left(\frac{(n-2)^2}{S_n^2 - (n-2)}, \frac{(n-2)}{S_n^2}\right)$$

Generating marginal probabilities

Correlation matrices Marginal probablities were calculated using the following data generating model:

$$logit(P(y_{ijk} = 1)) = \alpha + \beta_j + \theta X_{ij}$$

where y_{ijk} is the outcome of individual $k = 1, ..., K_{ij}$, in cluster $i = i, ..., C$ in measurement occasion $j = 1, ..., 6$. Note that there were 6 measurement occasions regardless of the trial design number of sequences. Designs with three sequences has 2 measurement occasions within each trial period.

α is the log odds of the outcome in the control condition in the first measurement occasion

β_j is the log odds ratio comparing the outcome in jth measurement occasion to the first measurement occasion, hence $\beta_1 = 0$.

θ is the intervention effect.

X_{ij} is an indicator equal to 1 if cluster i received the intervention for measurement occasion j and 0 otherwise. In the design with three sequences, X_{ij} is the same in measurement occasions 1 and 2, 3 and 4, and 5 and 6.

Data were generated so that observations in the same cluster were correlated to one another. For each cluster, correlations were simulated as defined in the correlation matrix below:

$$R_{lk} = \rho r_0^{|j_k - j_l|} r_1^{|X_{ij_k} - X_{ij_l}|} \mathbf{J} + (1-\rho)\mathbf{I}$$

where $\mathbf{J}$ is an m_i x m_i matrix of ones, $\mathbf{I}$ is an m_i x m_i identity matrix, m_i is the size of each cluster, j_k is the measurement occasion containing observation k, ρ is the correlation of an observation with another observation in the same cluster and same measurement occasion (the ICC), r_0 is the reduction in correletion between observations in the same cluster with each successive measurement occasion, and r_1 is the reduction in correlation between observations where one is in the control condition and one is in the intervention condition. r_0 and r_1 took the following values in each of the four correlation scenarios used in this study:

Exchangeable: $r_0 = 1$ and $r_1 = 1$

Autocorrelated r= 0.6: $r_0 = 0.6$ and $r_1 = 1$

Autocorrelated r= 0.8: $r_0 = 0.8$ and $r_1 = 1$

Autocorrelated r= 0.8 with reduced correlation between observations in different intervention conditions: $r_0 = 0.8$ and $r_1 = 0.5$

Example In the scenario with 24 observations per cluster with common cluster size so that there are 4 observations per observation occasion, and take a trial design with three sequences. The first row of the correlation matrix corresponds to the correlation of the first observation in the first period of a cluster with each other observation within the cluster.

A cluster in the first sequence (see figure one) will have a correlation matrix with the first line :

$$\left(\begin{bmatrix} 1 \\ \rho \\ \rho \\ \rho \end{bmatrix}^T, \begin{bmatrix} \rho r_0 \\ \rho r_0 \\ \rho r_0 \\ \rho r_0 \end{bmatrix}^T, \begin{bmatrix} \rho r_0^2 \\ \rho r_0^2 \\ \rho r_0^2 \\ \rho r_0^2 \end{bmatrix}^T, \begin{bmatrix} \rho r_0^3 \\ \rho r_0^3 \\ \rho r_0^3 \\ \rho r_0^3 \end{bmatrix}^T, \begin{bmatrix} \rho r_0^4 \\ \rho r_0^4 \\ \rho r_0^4 \\ \rho r_0^4 \end{bmatrix}^T, \begin{bmatrix} \rho r_0^5 \\ \rho r_0^5 \\ \rho r_0^5 \\ \rho r_0^5 \end{bmatrix}^T \right)$$

A cluster in the second sequence will have a correlation matrix with the first line:

$$\left(\begin{bmatrix} 1 \\ \rho \\ \rho \\ \rho \end{bmatrix}^T, \begin{bmatrix} \rho r_0 \\ \rho r_0 \\ \rho r_0 \\ \rho r_0 \end{bmatrix}^T, \begin{bmatrix} \rho r_0^2 r_1 \\ \rho r_0^2 r_1 \\ \rho r_0^2 r_1 \\ \rho r_0^2 r_1 \end{bmatrix}^T, \begin{bmatrix} \rho r_0^3 r_1 \\ \rho r_0^3 r_1 \\ \rho r_0^3 r_1 \\ \rho r_0^3 r_1 \end{bmatrix}^T, \begin{bmatrix} \rho r_0^4 r_1 \\ \rho r_0^4 r_1 \\ \rho r_0^4 r_1 \\ \rho r_0^4 r_1 \end{bmatrix}^T, \begin{bmatrix} \rho r_0^5 r_1 \\ \rho r_0^5 r_1 \\ \rho r_0^5 r_1 \\ \rho r_0^5 r_1 \end{bmatrix}^T \right)$$

A cluster in the third sequence will have a correlation matrix with the first line:

$$\left(\begin{bmatrix} 1 \\ \rho \\ \rho \\ \rho \end{bmatrix}^T, \begin{bmatrix} \rho r_0 \\ \rho r_0 \\ \rho r_0 \\ \rho r_0 \end{bmatrix}^T, \begin{bmatrix} \rho r_0^2 \\ \rho r_0^2 \\ \rho r_0^2 \\ \rho r_0^2 \end{bmatrix}^T, \begin{bmatrix} \rho r_0^3 \\ \rho r_0^3 \\ \rho r_0^3 \\ \rho r_0^3 \end{bmatrix}^T, \begin{bmatrix} \rho r_0^4 r_1 \\ \rho r_0^4 r_1 \\ \rho r_0^4 r_1 \\ \rho r_0^4 r_1 \end{bmatrix}^T, \begin{bmatrix} \rho r_0^5 r_1 \\ \rho r_0^5 r_1 \\ \rho r_0^5 r_1 \\ \rho r_0^5 r_1 \end{bmatrix}^T \right)$$

Sampling mechanism Using these probabilities and correlation matrices, we generated data using the methods described by Emrich and Piedmonte [10] as follows:

1. The algorithm described by Emrish nd Piedmonte [10] is used to convert the binary correlation matrix into a covariance matrix for simulating data from a normal distribution.

2. A multivariate normal distribution, with zero means and the converted covariance matrix is used to generate correlated standard normal variables for observations in each cluster.
3. The probability of the binary outcome is converted into quantiles of a standard normal distribution (e.g. a probability of 50% leads to a value of 0)
4. The generated correlated continuous outcomes produced in step 2 are dichotomised based on the normal quantiles produced in step 3, to give the simulated binary data.

The code for this simulation study will be made available on github at https://github.com/jenniferthompson1/SW-CRT-GEE

Implementation of analysis methods

Generalised estimating equations were implemented with the package geepack [11]. We used the geesmv package [12] to implement all standard errors and to estimate Pan and Wall degrees of freedom. We used the saws package [5] to implement the Fay and Graubard degrees of freedom, with the d5 option. Both saws and geesmv are designed to be used with the package gee, and some small adaptations were required for use with geepack. Details of these adaptations are availabe on request.

Analysis of results

A small number of corrections resulted in very large standard errors. Standard errors larger than 5 (for log OR=0.26) were excluded from all further analyses and treated as methods that did not converge. Sensitivity analyses showed that results were similar when excluding results with different cut-off values (results not shown).

As well as graphical exploration of the simulation study data, we also used linear regression models to explore the association between scenario characteristics and analysis-method performance measures. The linear regression models included each simulation scenario characteristic listed in main table 1 and likelihood ratio tests were used to test for association between each characteristic and the outcome. Supplementary tables 1 - 7 shows the result of these regression analyses. Comparisons on power presented in the paper are marginal means estimated from such a regression model to deal with confounding by the subset of scenarios selected for 300 observations per cluster.

Supplementary text references

1. Liang KY and Zeger SL. Longitudinal Data-Analysis Using Generalized Linear-Models. Biometrika 1986; 73: 13-22
2. Kauermann G and Carroll RJ. A note on the efficiency of sandwich covariance matrix estimation. J Am Stat Assoc 2001; 96: 1387-1396
3. Klema V and Laub A. The singular value decomposition: Its computation and some applications. IEEE Transactions on automatic control 1980; 25(2): 164-176
4. Gallis JA, Li F and Turner EL. XTGEEBCV: Stata module to compute bias-corrected (small-sample) standard errors for generalized estimating equations. Stata Journal 2020; 20(2): 363-381
5. Fay M P and Graubard B I.Small-sample adjustments for Wald-type tests using sandwich estimators. Biometrics 2001; 57: 1198-206
6. Mancl LA and DeRouen TA. A covariance estimator for GEE with improved small-sample properties. Biometrics 2001; 57: 126-134
7. Morel JG, Bokossa MC and Neerchal NK. Small sample correction for the variance of GEE estimators. Biometrical Journal 2003; 45: 395-409

8. Mackinnon JG and White H. Some Heteroskedasticity-Consistent Covariance-Matrix Estimators with Improved Finite-Sample Properties. Journal of Econometrics 1985; 29: 305-325

9. Pan W and Wall MM. Small-sample adjustments in using the sandwich variance estimator in generalized estimating equations. Stat Med 2002; 21: 1429-1441

10. Emrich L J and Piedmonte M R. A Method for Generating High-Dimensional Multivariate Binary Variates. The American Statistician 1991; 45: 302-304

11. Højsaard S, Halekoh U & YAan J The R Package geepack for Generalized Estimating Equations Journal of Statistical Software 2006; 15(2): 1-11

12. Wang M geesmv: Modified Variance Estimators for Generalized Estimating Equations. R package version 1.3. 2015.

Supplementary Table 1: Phase one factors associated with intervention effect estimate standardised bias

Characteristic	Effect	P value
Intercept	1.5 (0, 2.9)	0.05
Correlation structure		0.07
Exchangeable	0	
AR r=0.6	0.6 (-0.8, 2.1)	
AR r=0.8	-1.3 (-2.7, 0.2)	
Reduced intervention	-0.4 (-1.9, 1)	
ICC		0.89
0.01	0	
0.1	0.1 (-0.9, 1.1)	
Mean cluster size		0.47
24	0	
60	0.4 (-0.6, 1.4)	
Sequences		0.03
3	0	
6	1.1 (0.1, 2.1)	
Varying cluster size		0.12
No	0	
Yes	-0.8 (-1.8, 0.2)	

Supplementary Table 2: Phase one factors associated with relative error in standard errors of each correction

Standard error correction: Uncorr

	Independent		Exchangeable	
Characteristics	Effect	P value	Effect	P value
Intercept	-5.3 (-6.8, -3.8)	<0.001	-9.2 (-10.9, -7.4)	<0.001
Correlation structure		0.73		0.47
Exchangeable	0		0	
AR r=0.6	0 (-1.5, 1.5)		-0.5 (-2.2, 1.2)	
AR r=0.8	-0.7 (-2.2, 0.8)		0.9 (-0.9, 2.6)	
Reduced intervention	0.1 (-1.4, 1.6)		0.3 (-1.4, 2.1)	
ICC		<0.001		0.83
0.01	0		0	
0.1	-2 (-3.1, -1)		0.1 (-1.1, 1.4)	
Mean cluster size		0.97		0.97
24	0		0	
60	0 (-1.1, 1)		0 (-1.3, 1.2)	
Sequences		0.1		0.86
3	0		0	
6	-0.9 (-1.9, 0.2)		0.1 (-1.1, 1.3)	
Varying cluster size		<0.001		0.002
No	0		0	
Yes	-3.4 (-4.4, -2.3)		-2 (-3.2, -0.8)	

Standard error correction: KC

	Independent		Exchangeable	
Characteristics	Effect	P value	Effect	P value
Intercept	0.3 (-1.3, 1.9)	0.7	3.4 (-2.2, 9.1)	0.23
Correlation structure		0.74		<0.001
Exchangeable	0		0	
AR r=0.6	0 (-1.6, 1.6)		-13.6 (-19.2, -7.9)	
AR r=0.8	-0.7 (-2.3, 0.9)		-11.1 (-16.7, -5.5)	
Reduced intervention	0.1 (-1.5, 1.6)		-12.6 (-18.3, -7)	
ICC		0.004		<0.001
0.01	0		0	
0.1	-1.6 (-2.7, -0.5)		10.8 (6.8, 14.8)	
Mean cluster size		0.67		0.004
24	0		0	
60	0.2 (-0.9, 1.4)		5.8 (1.8, 9.8)	
Sequences		0.64		0.09
3	0		0	
6	-0.3 (-1.4, 0.8)		3.4 (-0.5, 7.4)	
Varying cluster size		0.18		0.37
No	0		0	
Yes	-0.8 (-1.9, 0.3)		1.8 (-2.2, 5.8)	

Standard error correction: FG

	Independent		Exchangeable	
Characteristics	Effect	P value	Effect	P value
Intercept	2.9 (1.2, 4.5)	0.001	-0.6 (-3, 1.9)	0.66
Correlation structure		0.27		0.57
Exchangeable	0		0	
AR r=0.6	-1.3 (-3, 0.3)		1.5 (-0.9, 4)	
AR r=0.8	-1.5 (-3.2, 0.1)		0.4 (-2.1, 2.8)	
Reduced intervention	-1 (-2.7, 0.6)		1.3 (-1.2, 3.7)	
ICC		0.69		<0.001
0.01	0		0	
0.1	-0.2 (-1.4, 0.9)		-4.6 (-6.3, -2.8)	
Mean cluster size		0.24		0.2
24	0		0	
60	0.7 (-0.5, 1.9)		-1.2 (-2.9, 0.6)	
Sequences		<0.001		0.006
3	0		0	
6	-2.8 (-4, -1.6)		-2.5 (-4.2, -0.7)	
Varying cluster size		0.02		0.05
No	0		0	
Yes	-1.4 (-2.5, -0.2)		1.7 (0, 3.5)	

Standard error correction: MD

	Independent		Exchangeable	
Characteristics	Effect	P value	Effect	P value
Intercept	6.2 (4.6, 7.9)	<0.001	4.8 (2.2, 7.5)	<0.001
Correlation structure		0.77		0.87
Exchangeable	0		0	
AR r=0.6	-0.1 (-1.8, 1.6)		0.6 (-2.1, 3.2)	
AR r=0.8	-0.8 (-2.4, 0.9)		0.1 (-2.5, 2.8)	
Reduced intervention	0 (-1.7, 1.7)		1 (-1.6, 3.6)	
ICC		0.09		<0.001
0.01	0		0	
0.1	-1 (-2.2, 0.2)		-3.6 (-5.4, -1.7)	
Mean cluster size		0.28		0.18
24	0		0	
60	0.7 (-0.5, 1.8)		-1.3 (-3.1, 0.6)	
Sequences		0.49		0.58
3	0		0	
6	0.4 (-0.8, 1.6)		0.5 (-1.3, 2.4)	
Varying cluster size		<0.001		<0.001
No	0		0	
Yes	2.8 (1.6, 4)		4.6 (2.7, 6.4)	

Standard error correction: MBN

	Independent		Exchangeable	
Characteristics	Effect	P value	Effect	P value
Intercept	11.7 (9.7, 13.7)	<0.001	8.4 (6.8, 10)	<0.001
Correlation structure		0.49		0.08
Exchangeable	0		0	
AR r=0.6	1.3 (-0.7, 3.3)		-1.9 (-3.5, -0.3)	
AR r=0.8	-0.1 (-2.2, 1.9)		-1.5 (-3.1, 0.1)	
Reduced intervention	0.6 (-1.4, 2.6)		-1.7 (-3.3, -0.1)	
ICC		<0.001		0.56
0.01	0		0	
0.1	-6 (-7.4, -4.5)		-0.3 (-1.5, 0.8)	
Mean cluster size		0.07		0.96
24	0		0	
60	-1.3 (-2.7, 0.1)		0 (-1.2, 1.1)	
Sequences		<0.001		<0.001
3	0		0	
6	5.4 (4, 6.9)		7.8 (6.7, 9)	
Varying cluster size		<0.001		<0.001
No	0		0	
Yes	-3.2 (-4.6, -1.8)		-2.8 (-4, -1.7)	

Standard error correction: MW

	Independent		Exchangeable	
Characteristics	Effect	P value	Effect	P value
Intercept	7.6 (5.8, 9.5)	<0.001	3.1 (1.5, 4.7)	<0.001
Correlation structure		0.78		0.68
Exchangeable	0		0	
AR r=0.6	0 (-1.8, 1.8)		-0.6 (-2.2, 1)	
AR r=0.8	-0.7 (-2.6, 1.1)		-0.1 (-1.6, 1.5)	
Reduced intervention	0.1 (-1.7, 1.9)		0.4 (-1.2, 2)	
ICC		<0.001		0.21
0.01	0		0	
0.1	-2.5 (-3.8, -1.2)		0.7 (-0.4, 1.9)	
Mean cluster size		0.89		0.34
24	0		0	
60	-0.1 (-1.4, 1.2)		0.5 (-0.6, 1.7)	
Sequences		<0.001		<0.001
3	0		0	
6	12.2 (10.9, 13.5)		12.6 (11.5, 13.7)	
Varying cluster size		<0.001		<0.001
No	0		0	
Yes	-4.1 (-5.4, -2.8)		-3 (-4.2, -1.9)	

Supplementary Table 3: Phase one degrees of freedom

Degrees of freedom: PW

	Independent		Exchangeable	
Characteristics	Effect	P value	Effect	P value
Intercept	62.6 (59.9, 65.2)	<0.001	53.3 (50.1, 56.5)	<0.001
Correlation structure		<0.001		<0.001
Exchangeable	0		0	
AR r=0.6	-5.6 (-8.2, -2.9)		7.2 (4, 10.4)	
AR r=0.8	-2.1 (-4.7, 0.5)		6 (2.8, 9.2)	
Reduced intervention	-3 (-5.7, -0.4)		5.4 (2.3, 8.6)	
ICC		<0.001		<0.001
0.01	0		0	
0.1	10.5 (8.6, 12.3)		-6.8 (-9.1, -4.5)	
Mean cluster size		<0.001		0.14
24	0		0	
60	3.3 (1.4, 5.1)		-1.7 (-4, 0.6)	
Sequences		<0.001		<0.001
3	0		0	
6	-15.2 (-17.1, -13.4)		-12.6 (-14.8, -10.3)	
Varying cluster size		<0.001		0.002
No	0		0	
Yes	4.2 (2.4, 6.1)		3.7 (1.4, 5.9)	

Degrees of freedom: FG

Characteristics	Independent		Exchangeable	
	Effect	P value	Effect	P value
Intercept	14.4 (14.3, 14.6)	<0.001	14.3 (14, 14.5)	<0.001
Correlation structure		0.97		<0.001
Exchangeable	0		0	
AR r=0.6	0 (-0.1, 0.2)		-0.6 (-0.8, -0.3)	
AR r=0.8	0 (-0.1, 0.1)		-0.3 (-0.5, -0.1)	
Reduced intervention	0 (-0.1, 0.2)		-0.4 (-0.6, -0.1)	
ICC		0.04		<0.001
0.01	0		0	
0.1	-0.1 (-0.2, 0)		2.1 (2, 2.3)	
Mean cluster size		<0.001		<0.001
24	0		0	
60	-0.2 (-0.3, -0.1)		0.4 (0.2, 0.6)	
Sequences		<0.001		0.002
3	0		0	
6	-0.5 (-0.6, -0.4)		-0.3 (-0.4, -0.1)	
Varying cluster size		<0.001		<0.001
No	0		0	
Yes	-3.6 (-3.7, -3.5)		-3.4 (-3.5, -3.2)	

Supplementary Table 4: Phase one factors associated with coverage of confidence intervals

Standard error correction: KC

	FG				PW			
	Independent		Exchangeable		Independent		Exchangeable	
Characteristics	Effect	P value	Effect	P value	Effect	P value	Effect	P value
Intercept	95.4 (95, 95.9)	<0.001	95.6 (94.9, 96.2)	<0.001	94.1 (93.5, 94.7)	<0.001	94.2 (93.5, 95)	<0.001
Correlation structure		0.35		<0.001		0.71		<0.001
Exchangeable	0		0		0		0	
AR r=0.6	0.1 (-0.4, 0.5)		-1.6 (-2.2, -1)		0.1 (-0.4, 0.7)		-2.4 (-3.1, -1.6)	
AR r=0.8	-0.3 (-0.7, 0.2)		-1.1 (-1.7, -0.5)		-0.2 (-0.8, 0.4)		-1.6 (-2.3, -0.9)	
Reduced intervention	0.1 (-0.3, 0.5)		-1.2 (-1.8, -0.6)		0.1 (-0.5, 0.7)		-1.6 (-2.3, -0.9)	
ICC		0.02		<0.001		0.02		<0.001
0.01	0		0		0		0	
0.1	-0.4 (-0.7, -0.1)		1.4 (0.9, 1.8)		-0.5 (-0.9, -0.1)		2.3 (1.8, 2.8)	
Mean cluster size		0.89		0.02		0.6		0.005
24	0		0		0		0	
60	0 (-0.3, 0.3)		0.5 (0.1, 1)		-0.1 (-0.5, 0.3)		0.7 (0.2, 1.2)	
Sequences		0.15		0.19		0.002		0.67
3	0		0		0		0	
6	-0.2 (-0.5, 0.1)		0.3 (-0.1, 0.7)		-0.6 (-1, -0.2)		0.1 (-0.4, 0.6)	
Varying cluster size		0.4		0.19		<0.001		0.02
No	0		0		0		0	
Yes	0.1 (-0.2, 0.4)		0.3 (-0.1, 0.7)		-0.7 (-1.1, -0.3)		-0.6 (-1.1, -0.1)	

	C-P				CP-P			
	Independent		Exchangeable		Independent		Exchangeable	
Characteristics	Effect	P value	Effect	P value	Effect	P value	Effect	P value
Intercept	94.4 (93.9, 94.9)	<0.001	94.4 (93.8, 95.1)	<0.001	95.6 (95.1, 96)	<0.001	95.4 (94.9, 96)	<0.001
Correlation structure		0.58		<0.001		0.2		<0.001
Exchangeable	0		0		0		0	
AR r=0.6	0.2 (-0.3, 0.7)		-2.1 (-2.8, -1.5)		0 (-0.5, 0.4)		-1.8 (-2.4, -1.3)	
AR r=0.8	-0.2 (-0.7, 0.4)		-1.5 (-2.2, -0.9)		-0.4 (-0.8, 0.1)		-1.4 (-1.9, -0.8)	
Reduced intervention	0.1 (-0.5, 0.6)		-1.6 (-2.2, -0.9)		0.1 (-0.4, 0.5)		-1.3 (-1.9, -0.8)	
ICC		0.007		<0.001		0.04		<0.001
0.01	0		0		0		0	
0.1	-0.5 (-0.9, -0.1)		2.4 (1.9, 2.8)		-0.3 (-0.7, 0)		1.9 (1.5, 2.3)	
Mean cluster size		0.36		<0.001		0.44		0.001
24	0		0		0		0	
60	-0.2 (-0.5, 0.2)		0.8 (0.4, 1.3)		-0.1 (-0.5, 0.2)		0.6 (0.3, 1)	
Sequences		0.58		0.008		0.36		<0.001
3	0		0		0		0	
6	-0.1 (-0.5, 0.3)		0.6 (0.2, 1.1)		0.2 (-0.2, 0.5)		0.7 (0.3, 1.1)	
Varying cluster size		0.01		0.14		<0.001		0.01
No	0		0		0		0	
Yes	-0.5 (-0.9, -0.1)		-0.3 (-0.8, 0.1)		-0.6 (-0.9, -0.3)		-0.5 (-0.9, -0.1)	

	CP-C-P			
	Independent		Exchangeable	
Characteristics	Effect	P value	Effect	P value
Intercept	94.4 (93.8, 94.9)	<0.001	94.5 (93.8, 95.2)	<0.001
Correlation structure		0.71		<0.001
Exchangeable	0		0	
AR r=0.6	0.2 (-0.4, 0.7)		-2.3 (-3, -1.6)	
AR r=0.8	-0.1 (-0.6, 0.4)		-1.5 (-2.2, -0.9)	
Reduced intervention	0.1 (-0.4, 0.7)		-1.6 (-2.3, -0.9)	
ICC		0.01		<0.001
0.01	0		0	
0.1	-0.5 (-0.9, -0.1)		2.2 (1.8, 2.7)	
Mean cluster size		0.69		0.004
24	0		0	
60	-0.1 (-0.5, 0.3)		0.7 (0.2, 1.2)	
Sequences		<0.001		0.79
3	0		0	
6	-0.9 (-1.3, -0.6)		-0.1 (-0.6, 0.4)	
Varying cluster size		0.001		0.01
No	0		0	
Yes	-0.6 (-1, -0.2)		-0.6 (-1.1, -0.1)	

Standard error correction: FG

	FG				PW			
	Independent		Exchangeable		Independent		Exchangeable	
Characteristics	Effect	P value	Effect	P value	Effect	P value	Effect	P value
Intercept	95.9 (95.5, 96.3)	<0.001	95.1 (94.7, 95.6)	<0.001	94.6 (94, 95.1)	<0.001	93.7 (93.1, 94.3)	<0.001
Correlation structure		0.07		0.59		0.81		0.49
Exchangeable	0		0		0		0	
AR r=0.6	-0.2 (-0.6, 0.2)		0.1 (-0.3, 0.6)		-0.1 (-0.6, 0.5)		-0.2 (-0.8, 0.3)	
AR r=0.8	-0.5 (-0.9, -0.1)		0.1 (-0.3, 0.6)		-0.2 (-0.8, 0.3)		0.1 (-0.5, 0.6)	
Reduced intervention	-0.1 (-0.5, 0.3)		0.3 (-0.1, 0.8)		0 (-0.5, 0.5)		0.2 (-0.4, 0.8)	
ICC		0.71		<0.001		0.81		0.06
0.01	0		0		0		0	
0.1	0.1 (-0.2, 0.3)		-0.8 (-1.1, -0.5)		0 (-0.4, 0.3)		-0.4 (-0.8, 0)	
Mean cluster size		0.98		0.41		0.88		0.45
24	0		0		0		0	
60	0 (-0.3, 0.3)		-0.1 (-0.4, 0.2)		0 (-0.4, 0.4)		-0.2 (-0.6, 0.2)	
Sequences		<0.001		0.02		<0.001		<0.001
3	0		0		0		0	
6	-0.6 (-0.9, -0.4)		-0.4 (-0.7, -0.1)		-1.2 (-1.6, -0.8)		-0.9 (-1.3, -0.5)	
Varying cluster size		0.75		0.27		<0.001		0.004
No	0		0		0		0	
Yes	0 (-0.2, 0.3)		0.2 (-0.1, 0.5)		-0.8 (-1.1, -0.4)		-0.6 (-1, -0.2)	

	C-P				CP-P			
	Independent		Exchangeable		Independent		Exchangeable	
Characteristics	Effect	P value	Effect	P value	Effect	P value	Effect	P value
Intercept	94.9 (94.4, 95.4)	<0.001	94 (93.5, 94.5)	<0.001	96 (95.6, 96.4)	<0.001	95.1 (94.7, 95.5)	<0.001
Correlation structure		0.56		0.85		0.07		0.37
Exchangeable	0		0		0		0	
AR r=0.6	-0.1 (-0.6, 0.4)		-0.2 (-0.7, 0.3)		-0.3 (-0.7, 0.2)		-0.2 (-0.6, 0.2)	
AR r=0.8	-0.4 (-0.9, 0.2)		-0.1 (-0.6, 0.4)		-0.5 (-1, -0.1)		-0.1 (-0.5, 0.3)	
Reduced intervention	-0.1 (-0.6, 0.4)		0 (-0.5, 0.5)		-0.1 (-0.5, 0.4)		0.1 (-0.3, 0.6)	
ICC		0.51		0.007		0.73		0.05
0.01	0		0		0		0	
0.1	-0.1 (-0.5, 0.2)		-0.5 (-0.9, -0.1)		-0.1 (-0.4, 0.3)		-0.3 (-0.6, 0)	
Mean cluster size		0.9		0.86		0.73		0.75
24	0		0		0		0	
60	0 (-0.4, 0.3)		0 (-0.4, 0.3)		-0.1 (-0.4, 0.3)		0 (-0.2, 0.3)	
Sequences		<0.001		0.35		0.07		0.55
3	0		0		0		0	
6	-0.6 (-1, -0.3)		-0.2 (-0.5, 0.2)		-0.3 (-0.6, 0)		0.1 (-0.2, 0.4)	
Varying cluster size		<0.001		0.08		<0.001		<0.001
No	0		0		0		0	
Yes	-0.7 (-1, -0.3)		-0.3 (-0.7, 0)		-0.7 (-1, -0.4)		-0.7 (-0.9, -0.4)	

	CP-C-P			
	Independent		Exchangeable	
Characteristics	Effect	P value	Effect	P value
Intercept	94.8 (94.3, 95.3)	<0.001	93.9 (93.4, 94.5)	<0.001
Correlation structure		0.86		0.53
Exchangeable	0		0	
AR r=0.6	0 (-0.5, 0.5)		-0.2 (-0.7, 0.4)	
AR r=0.8	-0.2 (-0.7, 0.3)		0.1 (-0.5, 0.6)	
Reduced intervention	0 (-0.5, 0.5)		0.3 (-0.3, 0.8)	
ICC		0.9		0.07
0.01	0		0	
0.1	0 (-0.4, 0.3)		-0.4 (-0.8, 0)	
Mean cluster size		0.77		0.54
24	0		0	
60	-0.1 (-0.4, 0.3)		-0.1 (-0.5, 0.3)	
Sequences		<0.001		<0.001
3	0		0	
6	-1.4 (-1.8, -1.1)		-1.1 (-1.5, -0.7)	
Varying cluster size		<0.001		0.005
No	0		0	
Yes	-0.7 (-1.1, -0.4)		-0.6 (-0.9, -0.2)	

Phase 2

Supplementary Table 5: Phase two factors associated with intervention effect estimate bias

Characteristic	Effect	P value
Intercept	0.4 (-0.2, 1)	0.21
Clusters		<0.001
54	0	
48	0.1 (-0.5, 0.7)	
42	0.1 (-0.5, 0.7)	
24	0.5 (-0.1, 1.1)	
18	1.2 (0.6, 1.8)	
12	0.8 (0.2, 1.4)	
6	1.8 (1.2, 2.4)	
Correlation structure		0.4
Exchangeable	0	
AR r=0.6	-0.1 (-0.6, 0.3)	
AR r=0.8	0 (-0.4, 0.5)	
Reduced intervention	-0.3 (-0.8, 0.1)	
ICC		0.13
0.01	0	
0.05	0.3 (-0.1, 0.7)	
0.1	0.4 (0, 0.8)	
Mean cluster size		0.01
24	0	
60	-0.5 (-0.8, -0.2)	
300	-0.1 (-1.2, 0.9)	
Sequences		0.004
3	0	
6	0.5 (0.2, 0.8)	
Varying cluster size		0.22
No	0	
Yes	0.2 (-0.1, 0.5)	

Supplementary Table 6: Phase two factors associated with relative error in standard errors of each correction

Standard error correction: Uncorr

	Independent		Exchangeable	
Characteristics	Effect	P value	Effect	P value
Intercept	-0.3 (-1, 0.4)	0.39	-2.8 (-3.5, -2.1)	<0.001
Clusters		<0.001		<0.001
54	0		0	
48	-0.1 (-0.8, 0.6)		-0.2 (-0.8, 0.5)	
42	-0.8 (-1.5, -0.1)		-0.9 (-1.6, -0.3)	
24	-3.7 (-4.4, -3.1)		-4.5 (-5.1, -3.8)	
18	-5.4 (-6.1, -4.7)		-6.8 (-7.5, -6.2)	
12	-9.6 (-10.2, -8.9)		-11.7 (-12.3, -11)	
6	-21.1 (-21.8, -20.4)		-26.7 (-27.3, -26)	
Correlation structure		0.02		<0.001
Exchangeable	0		0	
AR r=0.6	0.4 (-0.1, 0.9)		-0.4 (-0.9, 0.1)	
AR r=0.8	0 (-0.5, 0.5)		-0.5 (-1, 0)	
Reduced intervention	-0.4 (-1, 0.1)		-1.1 (-1.6, -0.6)	
ICC		<0.001		<0.001
0.01	0		0	
0.05	-0.4 (-0.8, 0.1)		1 (0.5, 1.4)	
0.1	-1.4 (-1.8, -0.9)		1 (0.6, 1.5)	
Mean cluster size		0.004		0.002
24	0		0	
60	-0.6 (-0.9, -0.2)		0.5 (0.2, 0.9)	
300	-1 (-2.3, 0.2)		1.5 (0.3, 2.7)	
Sequences		<0.001		0.36
3	0		0	
6	-0.7 (-1.1, -0.3)		-0.2 (-0.5, 0.2)	
Varying cluster size		<0.001		<0.001
No	0		0	
Yes	-3.1 (-3.4, -2.7)		-1.9 (-2.2, -1.5)	

Standard error correction: KC

	Independent		Exchangeable	
Characteristics	Effect	P value	Effect	P value
Intercept	0.7 (0, 1.4)	0.06	1.3 (-2.1, 4.7)	0.45
Clusters		<0.001		0.96
54	0		0	
48	0.3 (-0.4, 1)		0.2 (-3.1, 3.5)	
42	-0.1 (-0.7, 0.6)		-0.2 (-3.5, 3)	
24	-0.6 (-1.3, 0.1)		-0.9 (-4.2, 2.3)	
18	-0.4 (-1.1, 0.3)		-0.8 (-4.1, 2.5)	
12	-1.2 (-1.9, -0.5)		-1.1 (-4.3, 2.2)	
6	-2.3 (-3, -1.6)		-1.3 (-4.6, 2)	
Correlation structure		0.007		<0.001
Exchangeable	0		0	
AR r=0.6	0.4 (-0.1, 1)		-13.2 (-15.6, -10.7)	
AR r=0.8	0 (-0.6, 0.5)		-10 (-12.4, -7.5)	
Reduced intervention	-0.5 (-1, 0)		-13 (-15.5, -10.5)	
ICC		<0.001		<0.001
0.01	0		0	
0.05	-0.1 (-0.6, 0.3)		6.4 (4.3, 8.5)	
0.1	-1.1 (-1.5, -0.6)		14.5 (12.4, 16.6)	
Mean cluster size		0.11		<0.001
24	0		0	
60	-0.3 (-0.7, 0)		6.5 (4.7, 8.2)	
300	-0.9 (-2.2, 0.3)		81.5 (75.7, 87.4)	
Sequences		0.48		<0.001
3	0		0	
6	-0.1 (-0.5, 0.2)		3.4 (1.7, 5.2)	
Varying cluster size		<0.001		0.002
No	0		0	
Yes	-0.9 (-1.3, -0.6)		2.8 (1, 4.5)	

Standard error correction: FG

	Independent		Exchangeable	
Characteristics	Effect	P value	Effect	P value
Intercept	2.2 (1.4, 3.1)	<0.001	1 (-0.8, 2.8)	0.28
Clusters		<0.001		0.005
54	0		0	
48	0.3 (-0.5, 1.1)		0.2 (-1.5, 2)	
42	0 (-0.8, 0.8)		-0.4 (-2.1, 1.4)	
24	-0.1 (-0.9, 0.6)		-1.6 (-3.3, 0.2)	
18	0.4 (-0.4, 1.2)		-1.9 (-3.7, -0.1)	
12	0.8 (0, 1.5)		-2.2 (-4, -0.5)	
6	4.5 (3.7, 5.2)		0.5 (-1.2, 2.3)	
Correlation structure		<0.001		0.14
Exchangeable	0		0	
AR r=0.6	-1.1 (-1.7, -0.5)		1.4 (0.1, 2.8)	
AR r=0.8	-1 (-1.6, -0.4)		0.3 (-1.1, 1.6)	
Reduced intervention	-1.8 (-2.4, -1.2)		0.1 (-1.2, 1.5)	
ICC		0.007		<0.001
0.01	0		0	
0.05	0.8 (0.3, 1.3)		-4.3 (-5.5, -3.2)	
0.1	0.5 (0, 1)		-5.6 (-6.8, -4.5)	
Mean cluster size		0.37		0.04
24	0		0	
60	0.3 (-0.2, 0.7)		-0.9 (-1.9, 0)	
300	0.7 (-0.8, 2.1)		2.2 (-1, 5.3)	
Sequences		<0.001		0.005
3	0		0	
6	-2.9 (-3.3, -2.5)		-1.3 (-2.3, -0.4)	
Varying cluster size		<0.001		<0.001
No	0		0	
Yes	-1.1 (-1.5, -0.6)		2.8 (1.9, 3.8)	

Supplementary Table 7: Phase two factors associated with coverage of confidence intervals

Standard error correction: KC

	FG				C-P			
	Independent		Exchangeable		Independent		Exchangeable	
Characteristics	Effect	P value	Effect	P value	Effect	P value	Effect	P value
Intercept	95.1 (94.9, 95.3)	<0.001	95.5 (95.2, 95.8)	<0.001	95 (94.7, 95.2)	<0.001	95.2 (94.9, 95.5)	<0.001
Clusters		<0.001		<0.001		<0.001		<0.001
54	0		0		0		0	
48	-0.1 (-0.3, 0.1)		-0.1 (-0.4, 0.1)		-0.1 (-0.3, 0.2)		-0.1 (-0.4, 0.1)	
42	-0.1 (-0.3, 0.1)		-0.2 (-0.4, 0.1)		-0.1 (-0.3, 0.1)		-0.2 (-0.4, 0.1)	
24	0 (-0.2, 0.2)		-0.2 (-0.4, 0.1)		-0.1 (-0.3, 0.1)		-0.2 (-0.4, 0.1)	
18	0.2 (0, 0.4)		-0.1 (-0.3, 0.2)		0.2 (0, 0.4)		-0.1 (-0.4, 0.2)	
12	0.4 (0.2, 0.6)		0.1 (-0.2, 0.3)		1 (0.8, 1.2)		0.5 (0.2, 0.7)	
6	1.4 (1.2, 1.6)		1.2 (0.9, 1.4)		4.6 (4.4, 4.8)		3.5 (3.2, 3.7)	
Correlation structure		0.009		<0.001		0.03		<0.001
Exchangeable	0		0		0		0	
AR r=0.6	0.2 (0, 0.3)		-1.3 (-1.5, -1.1)		0.2 (0, 0.3)		-1.2 (-1.4, -1.1)	
AR r=0.8	0 (-0.2, 0.1)		-0.9 (-1.1, -0.7)		0 (-0.1, 0.2)		-0.8 (-1, -0.6)	
Reduced intervention	0 (-0.2, 0.1)		-1.3 (-1.5, -1.1)		-0.1 (-0.2, 0.1)		-1.2 (-1.4, -1.1)	
ICC		<0.001		<0.001		0.004		<0.001
0.01	0		0		0		0	
0.05	0 (-0.1, 0.1)		0.8 (0.6, 0.9)		0 (-0.1, 0.2)		1 (0.9, 1.2)	
0.1	-0.2 (-0.4, -0.1)		1.3 (1.1, 1.5)		-0.2 (-0.3, 0)		1.7 (1.5, 1.8)	
Mean cluster size		<0.001		<0.001		<0.001		<0.001
24	0		0		0		0	
60	-0.3 (-0.4, -0.2)		0.5 (0.3, 0.6)		-0.2 (-0.3, -0.1)		0.6 (0.4, 0.7)	
300	-0.6 (-0.9, -0.2)		2.8 (2.3, 3.3)		-0.5 (-0.8, -0.1)		2.7 (2.2, 3.2)	
Sequences		0.23		<0.001		<0.001		<0.001
3	0		0		0		0	
6	0.1 (0, 0.2)		0.5 (0.3, 0.6)		0.5 (0.4, 0.6)		0.8 (0.7, 1)	
Varying cluster size		0.12		<0.001		<0.001		<0.001
No	0		0		0		0	
Yes	0.1 (0, 0.2)		0.3 (0.1, 0.4)		-0.5 (-0.6, -0.4)		-0.3 (-0.4, -0.1)	

Standard error correction: FG

	FG				C-P			
	Independent		Exchangeable		Independent		Exchangeable	
Characteristics	Effect	P value	Effect	P value	Effect	P value	Effect	P value
Intercept	95.3 (95.1, 95.6)	<0.001	95.2 (94.9, 95.5)	<0.001	95.2 (95, 95.4)	<0.001	94.7 (94.5, 94.9)	<0.001
Clusters		<0.001		<0.001		<0.001		<0.001
54	0		0		0		0	
48	0 (-0.2, 0.2)		-0.2 (-0.4, 0.1)		-0.1 (-0.3, 0.1)		-0.2 (-0.4, 0)	
42	0 (-0.2, 0.2)		-0.1 (-0.4, 0.1)		-0.1 (-0.3, 0.1)		-0.2 (-0.4, 0)	
24	0.1 (-0.1, 0.3)		-0.3 (-0.5, 0)		0.1 (-0.1, 0.3)		-0.3 (-0.5, -0.1)	
18	0.4 (0.2, 0.6)		-0.3 (-0.5, 0)		0.4 (0.2, 0.6)		-0.2 (-0.4, 0)	
12	0.6 (0.4, 0.8)		0 (-0.3, 0.2)		1.4 (1.2, 1.6)		0.8 (0.6, 1)	
6	1.9 (1.7, 2.1)		1.8 (1.6, 2)		4.7 (4.5, 4.9)		4.8 (4.6, 5)	
Correlation structure		0.02		0.005		0.04		0.005
Exchangeable	0		0		0		0	
AR r=0.6	0 (-0.2, 0.1)		0.2 (0, 0.4)		0 (-0.2, 0.1)		0.1 (-0.1, 0.2)	
AR r=0.8	-0.2 (-0.3, 0)		0 (-0.1, 0.2)		-0.1 (-0.3, 0)		0 (-0.2, 0.1)	
Reduced intervention	-0.2 (-0.4, 0)		-0.1 (-0.3, 0.1)		-0.2 (-0.3, 0)		-0.2 (-0.4, 0)	
ICC		0.05		<0.001		0.23		0.85
0.01	0		0		0		0	
0.05	0.2 (0, 0.3)		-0.4 (-0.6, -0.3)		0.1 (0, 0.2)		0 (-0.1, 0.2)	
0.1	0 (-0.1, 0.2)		-0.6 (-0.8, -0.5)		0 (-0.1, 0.2)		0 (-0.1, 0.2)	
Mean cluster size		<0.001		<0.001		0.004		0.04
24	0		0		0		0	
60	-0.2 (-0.3, -0.1)		-0.4 (-0.5, -0.2)		-0.2 (-0.3, -0.1)		-0.1 (-0.2, 0)	
300	-0.2 (-0.6, 0.2)		0 (-0.5, 0.4)		-0.2 (-0.6, 0.2)		0.2 (-0.2, 0.6)	
Sequences		<0.001		0.15		0.02		<0.001
3	0		0		0		0	
6	-0.3 (-0.4, -0.2)		-0.1 (-0.2, 0)		0.1 (0, 0.2)		0.4 (0.3, 0.5)	
Varying cluster size		0.75		<0.001		<0.001		<0.001
No	0		0		0		0	
Yes	0 (-0.1, 0.1)		0.2 (0.1, 0.4)		-0.5 (-0.6, -0.4)		-0.3 (-0.4, -0.2)	

Supplementary Figure 1: Phase one intervention effect estimate bias

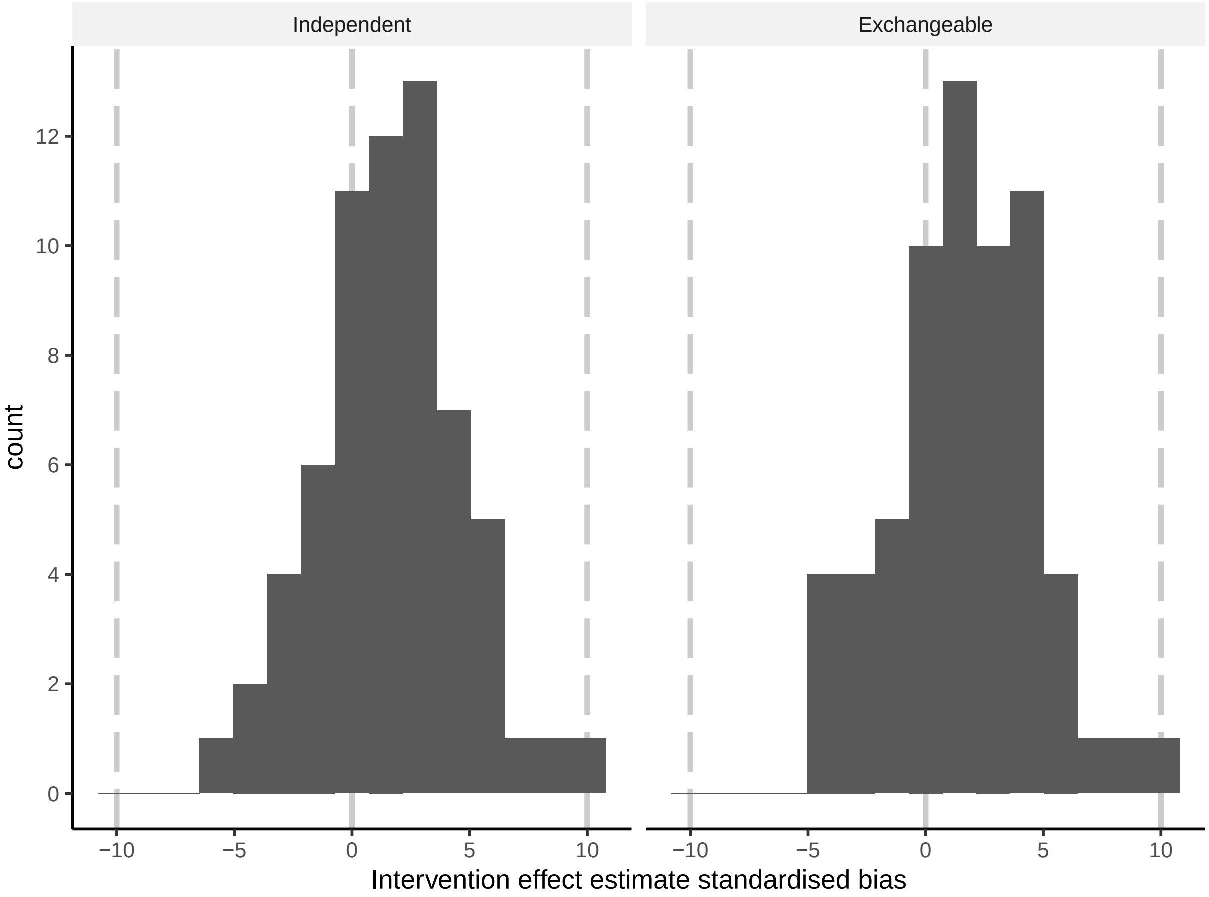

Supplementary Figure 2: Phase one relative error in standard errors by true correlation structure

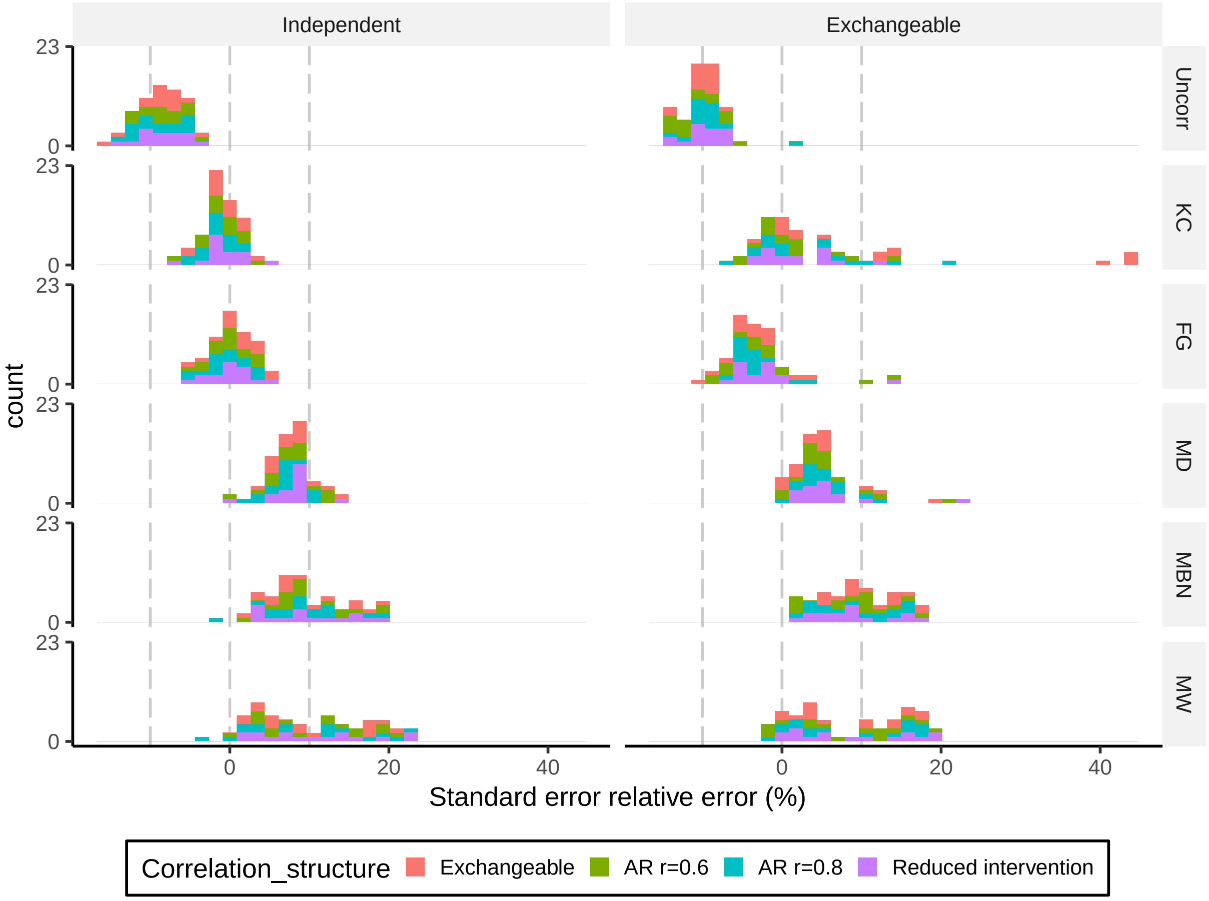

Supplementary Figure 3: Phase one relative error in standard errors by ICC

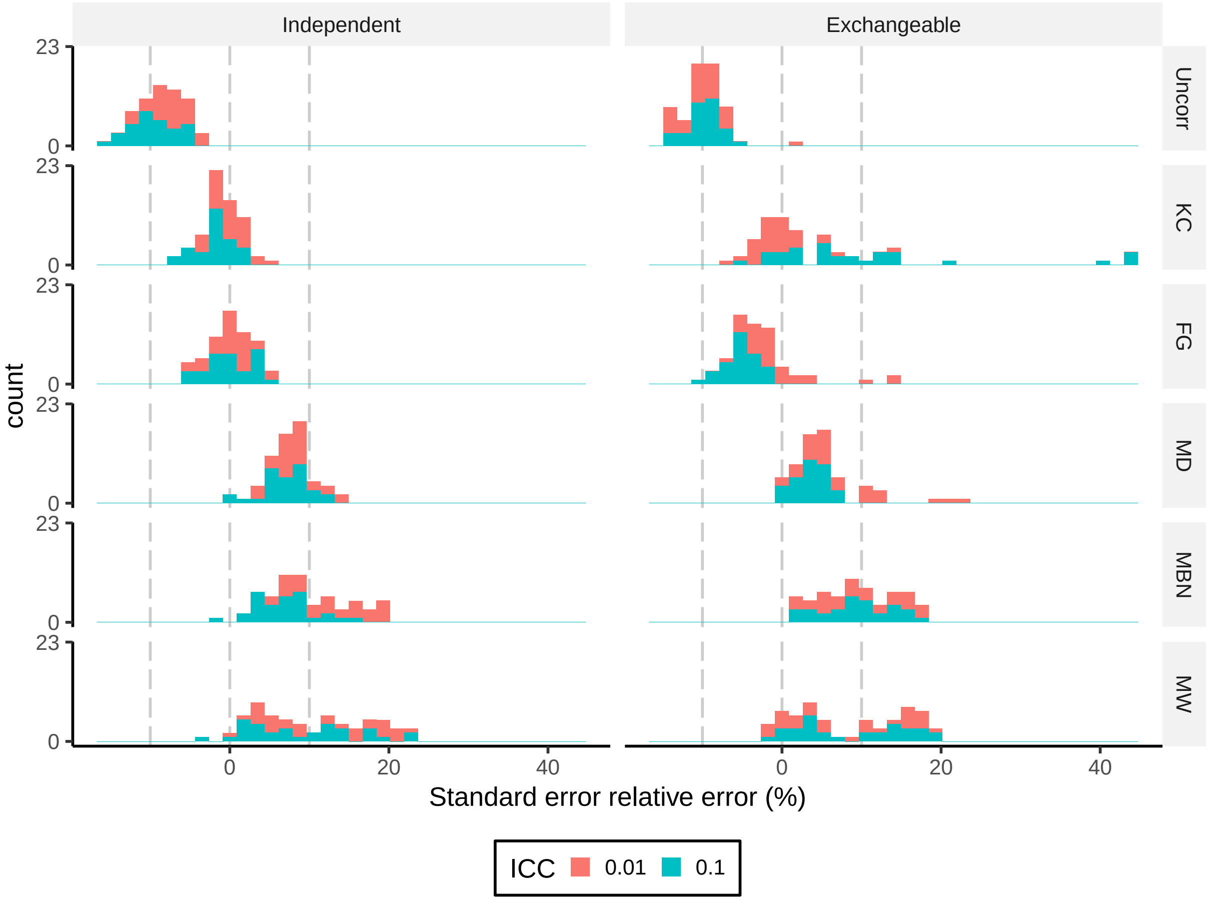

Supplementary Figure 4: Phase one relative error in standard errors by varying cluster size

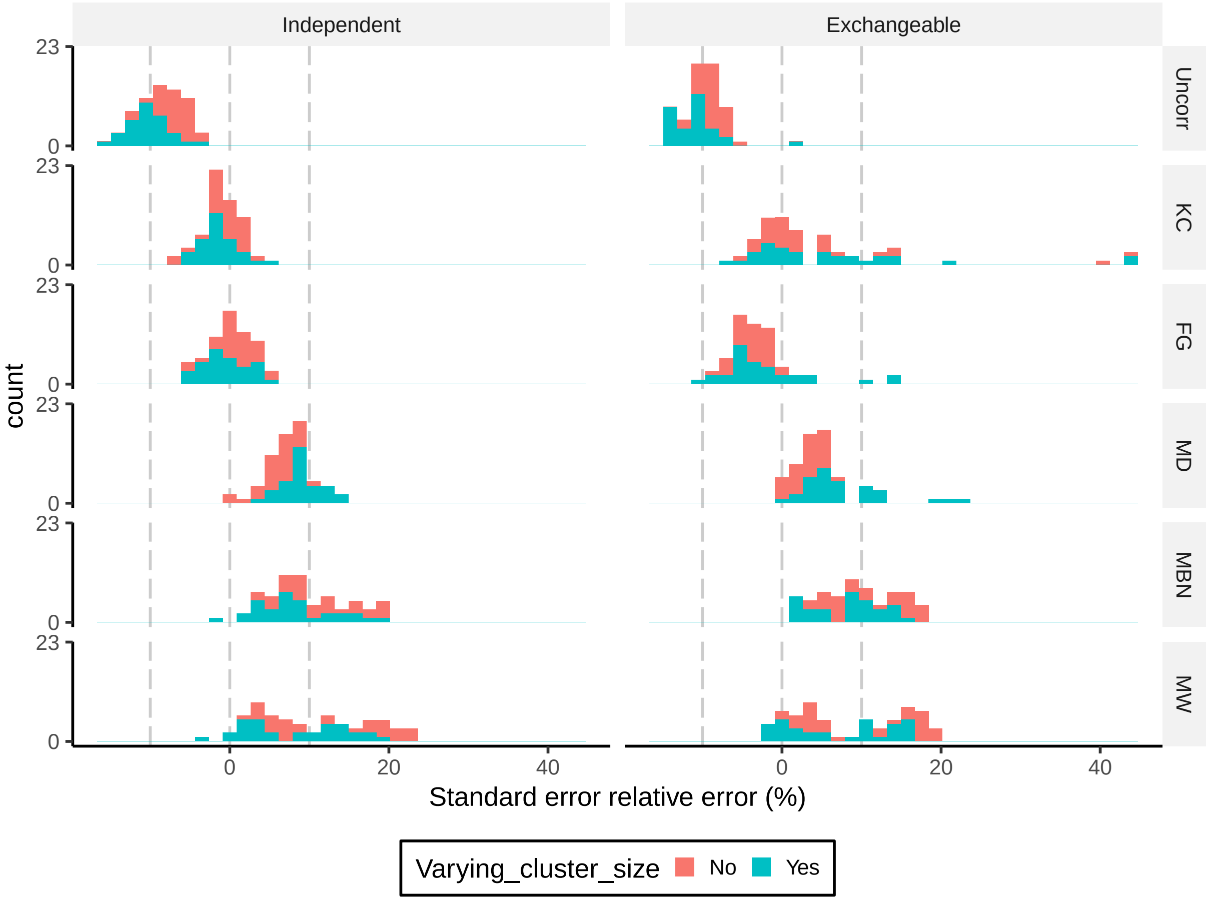

Supplementary Figure 5: Phase one relative error in standard errors by sequences

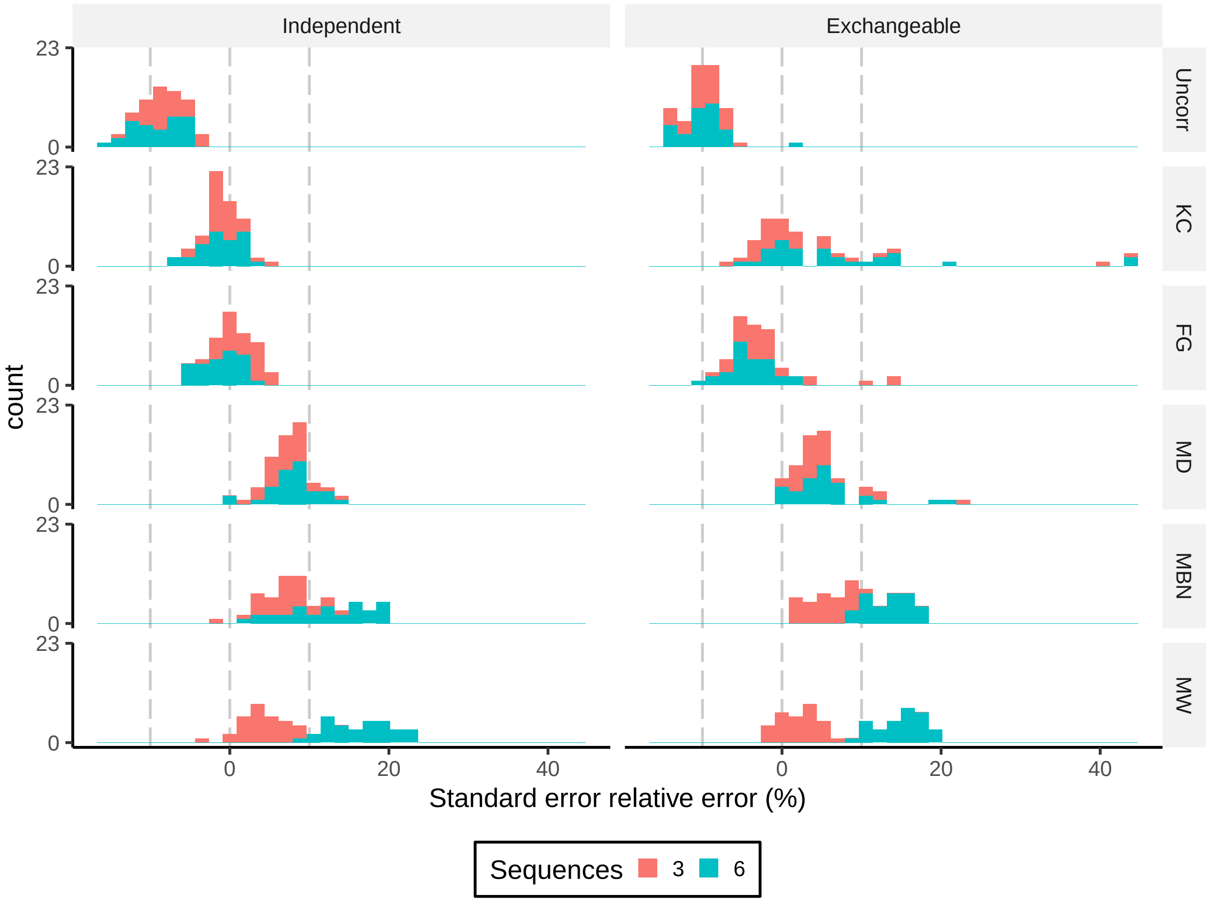

Supplementary Figure 6: Phase two intervention effect estimate bias

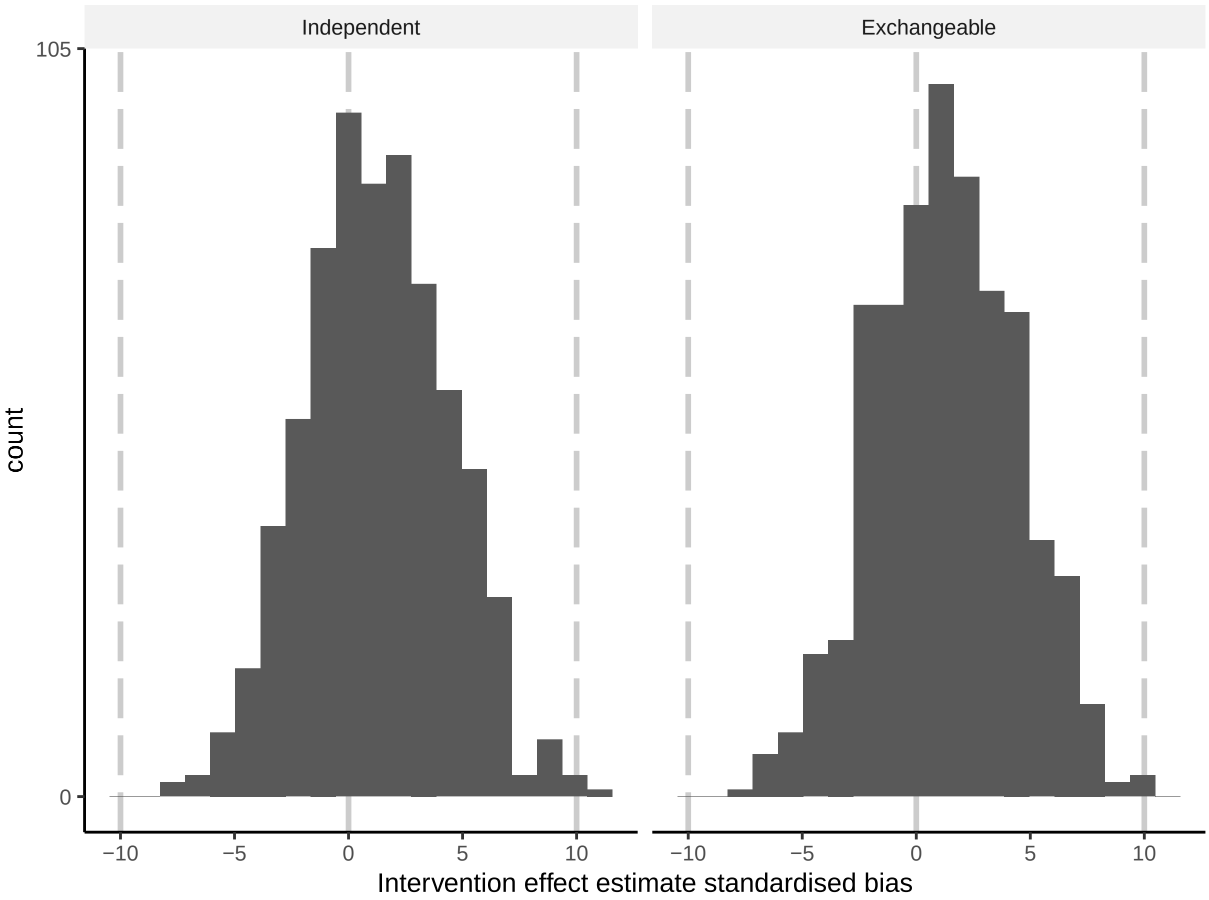

Supplementary Figure 7: Phase two relative error in FG and KC standard errors by ICC

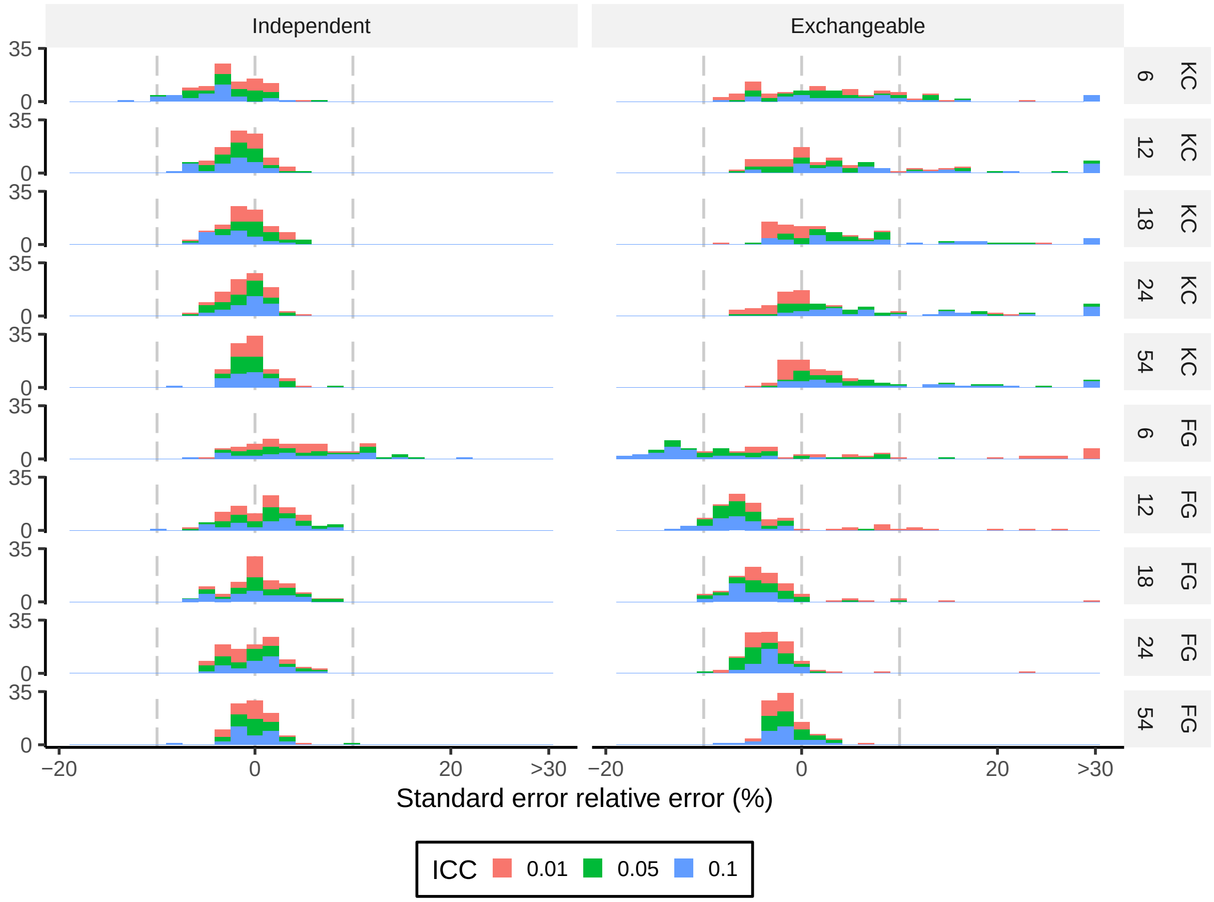

Supplementary Figure 8: Phase two relative error in uncorrected standard errors

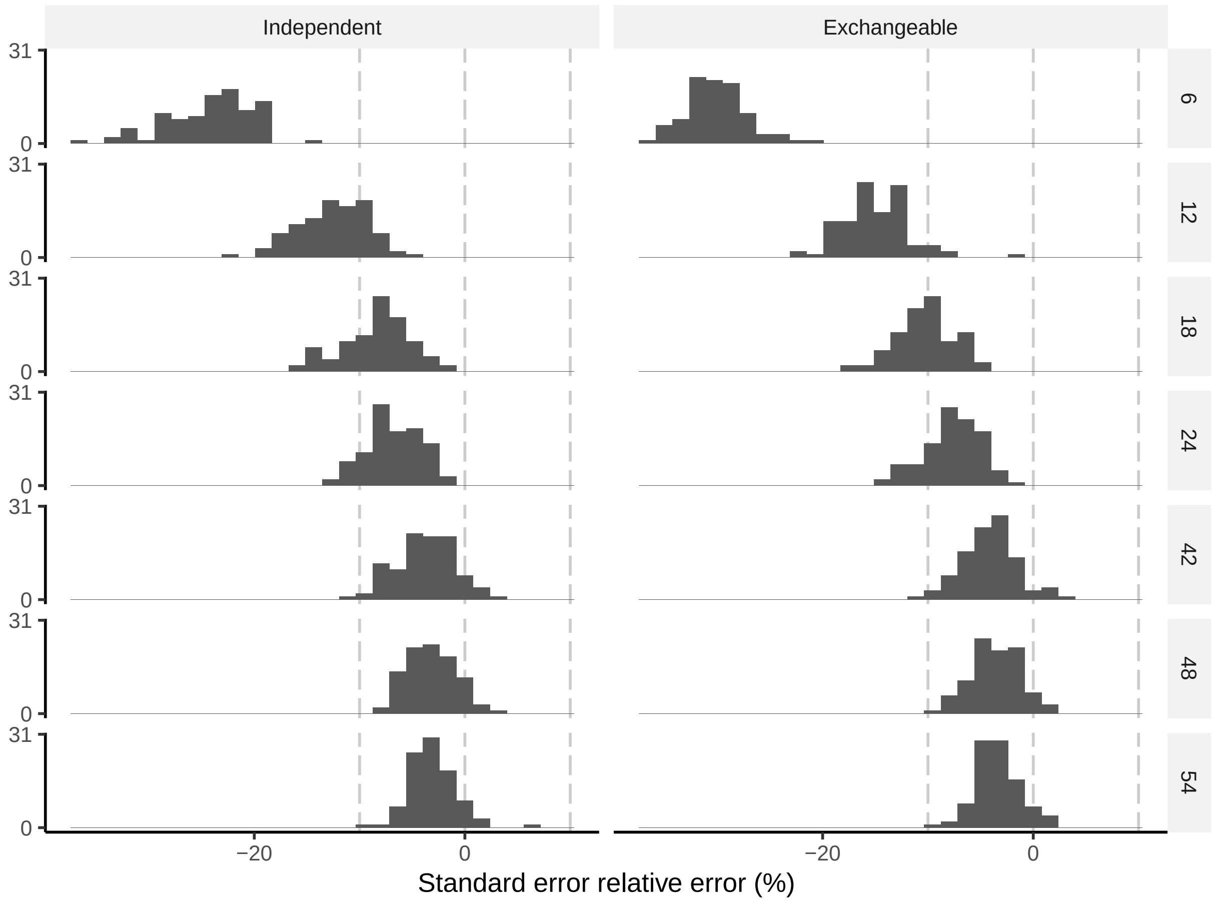

Supplementary Figure 9: Phase two difference in DF_{FG} and DF_{C-P}

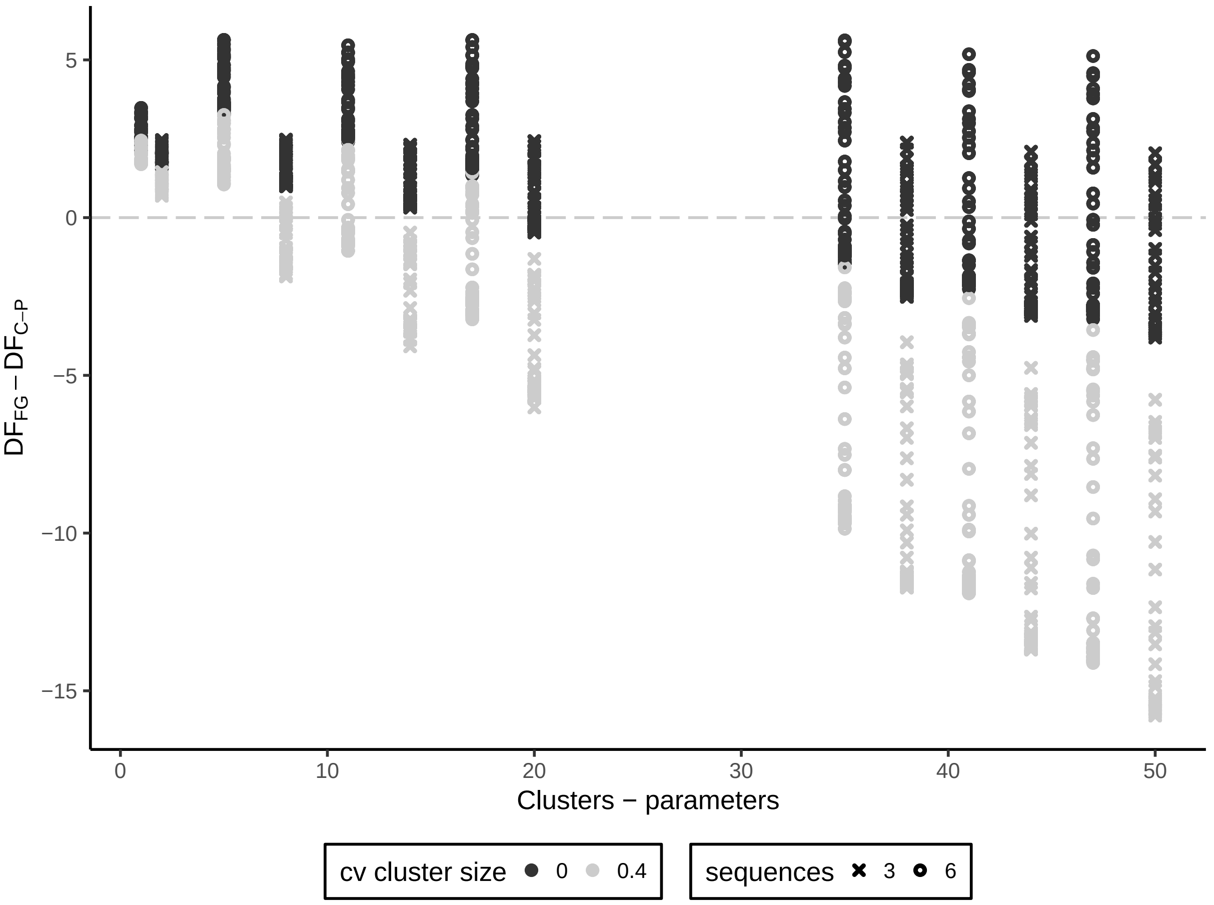

Supplementary Figure 10: Phase two confidence interval coverage of uncorrected standard errors

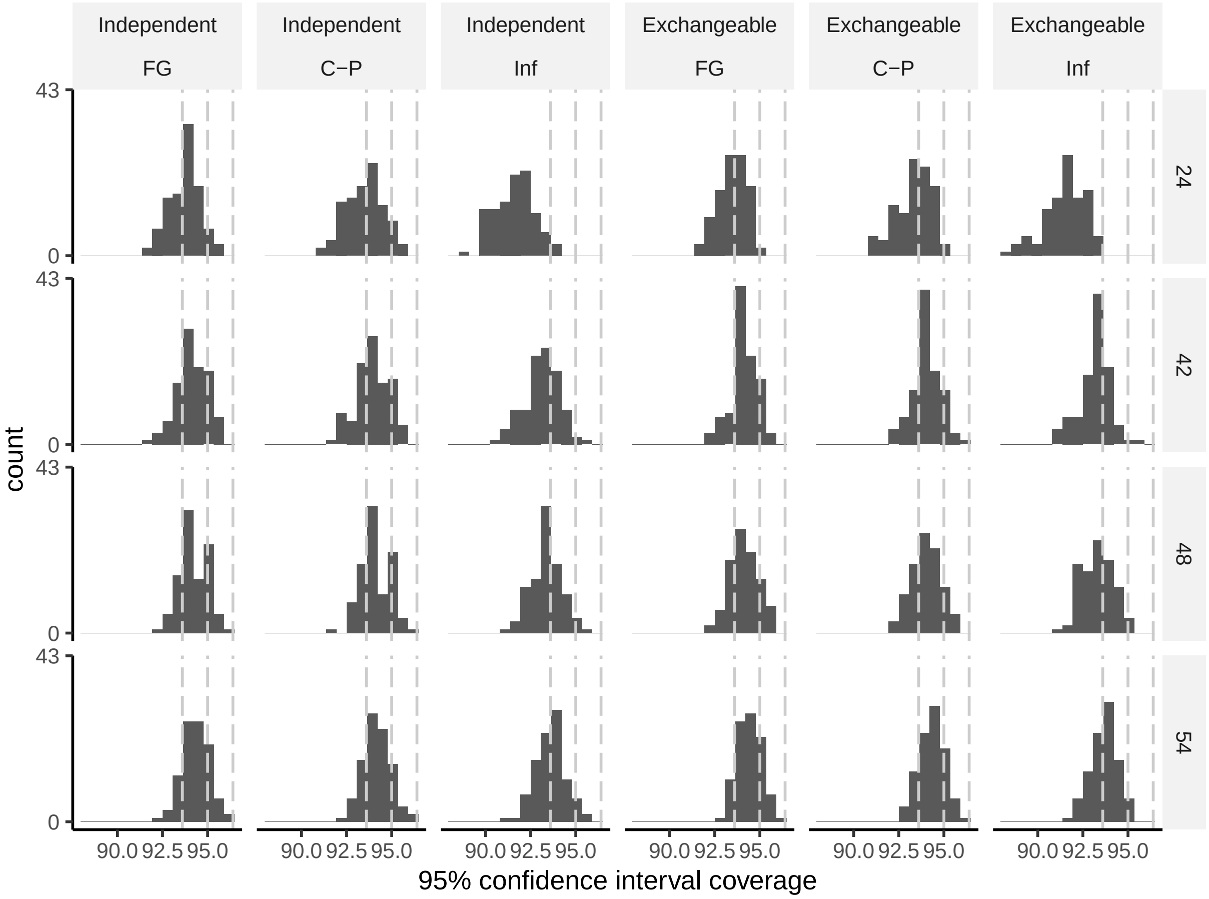

Supplementary Figure 11: Phase two comparison of power using FG and KC standard errors with an indpendent working correlation matrix

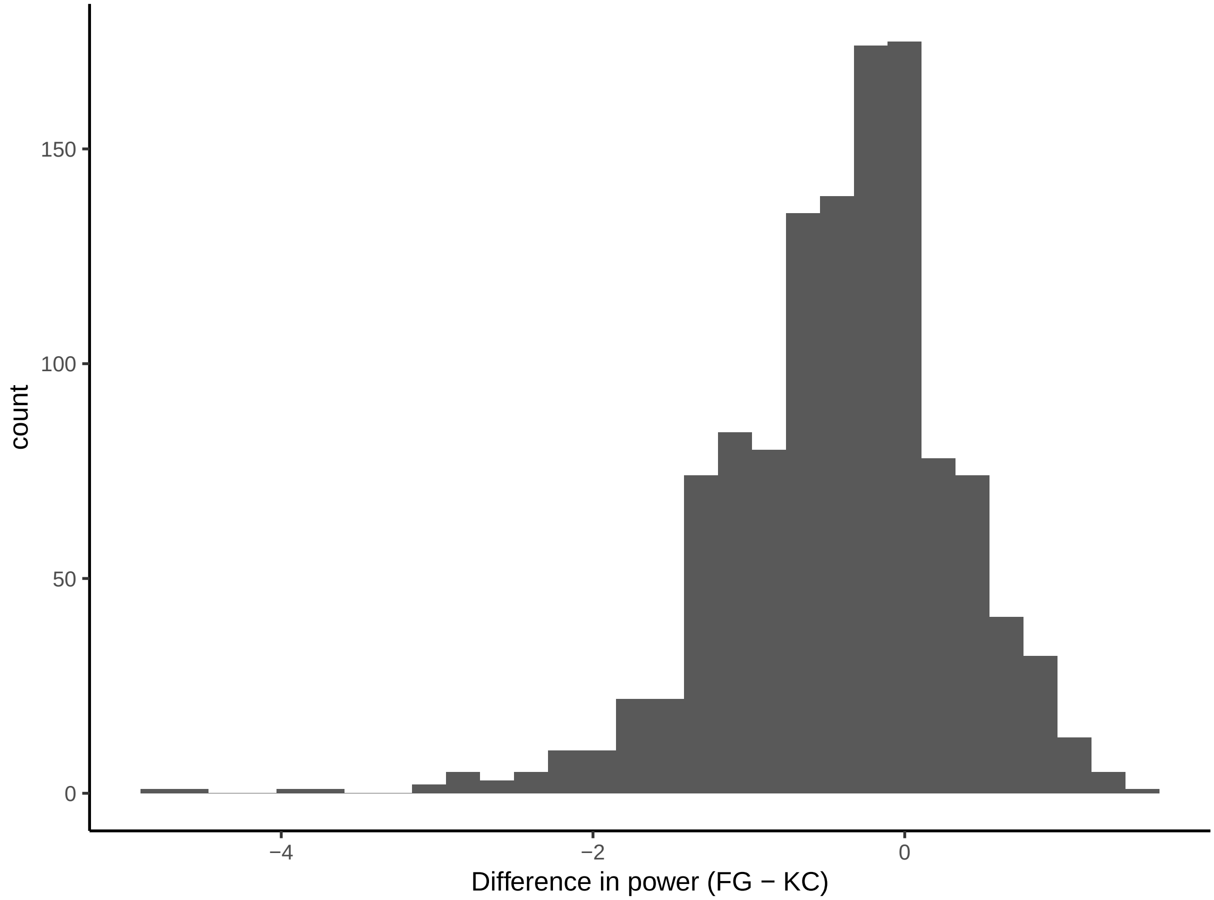

Supplementary Figure 12: Phase two comparison of power using DF_{FG} and DF_{C-P} with an indpendent working correlation matrix

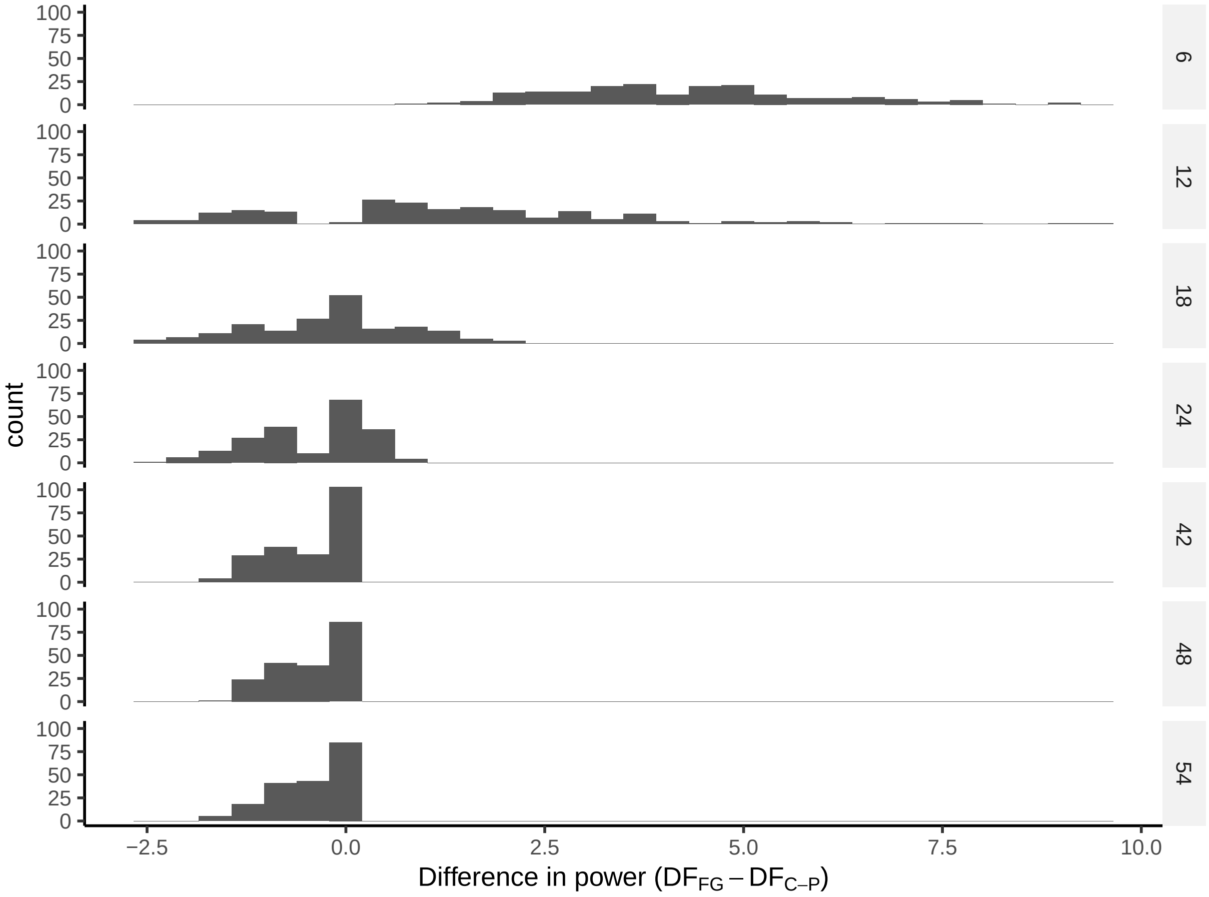

Supplementary Figure 13: Phase two comparison of power using an exchangeable and indpendent working correlation matrix with DF_{FG}

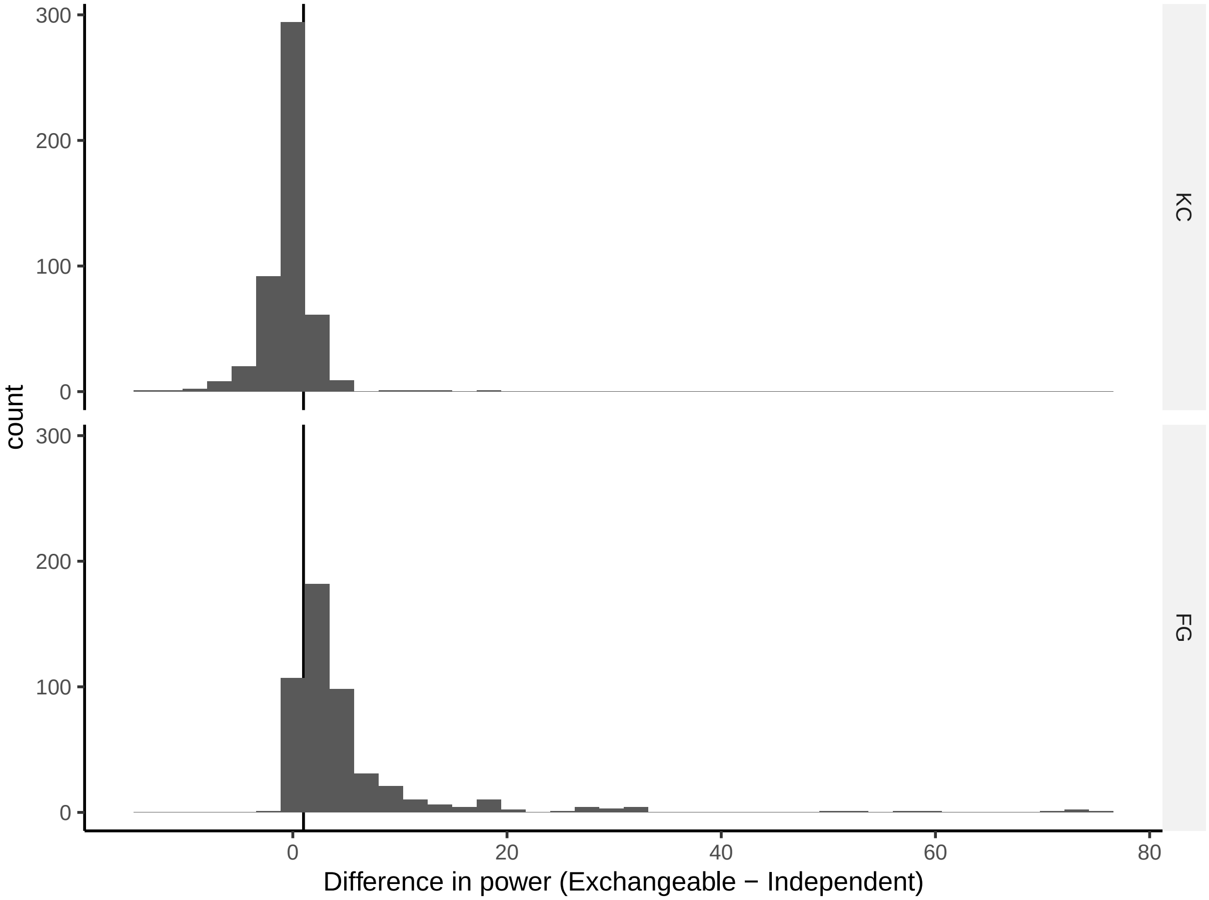

Supplementary Figure 14: Post hoc analysis: Relative error in KC-approximation standard errors in phase 2 scenarios

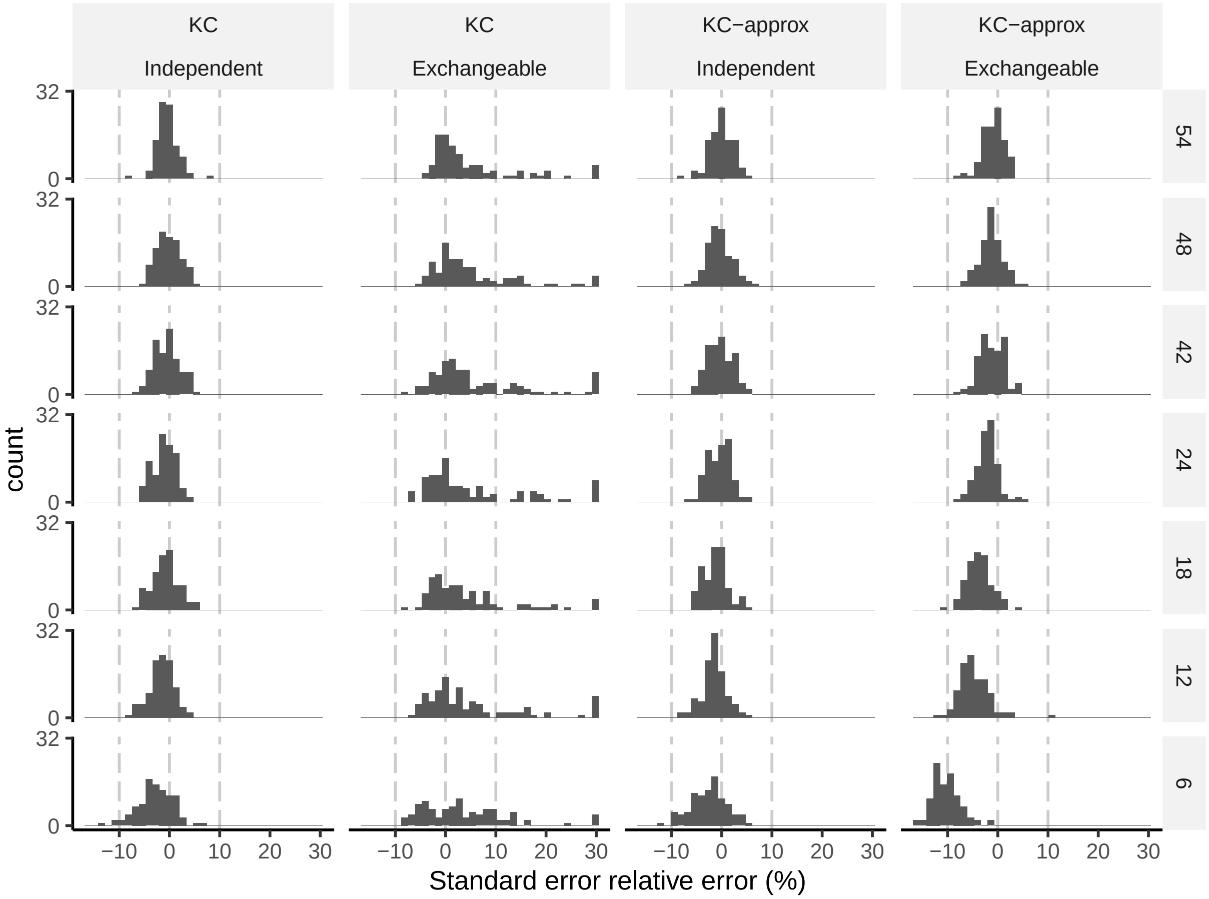

Supplementary Figure 15: Post hoc analysis: 95% confidence interval coverage with KC-approximation standard errors in phase 2 scenarios

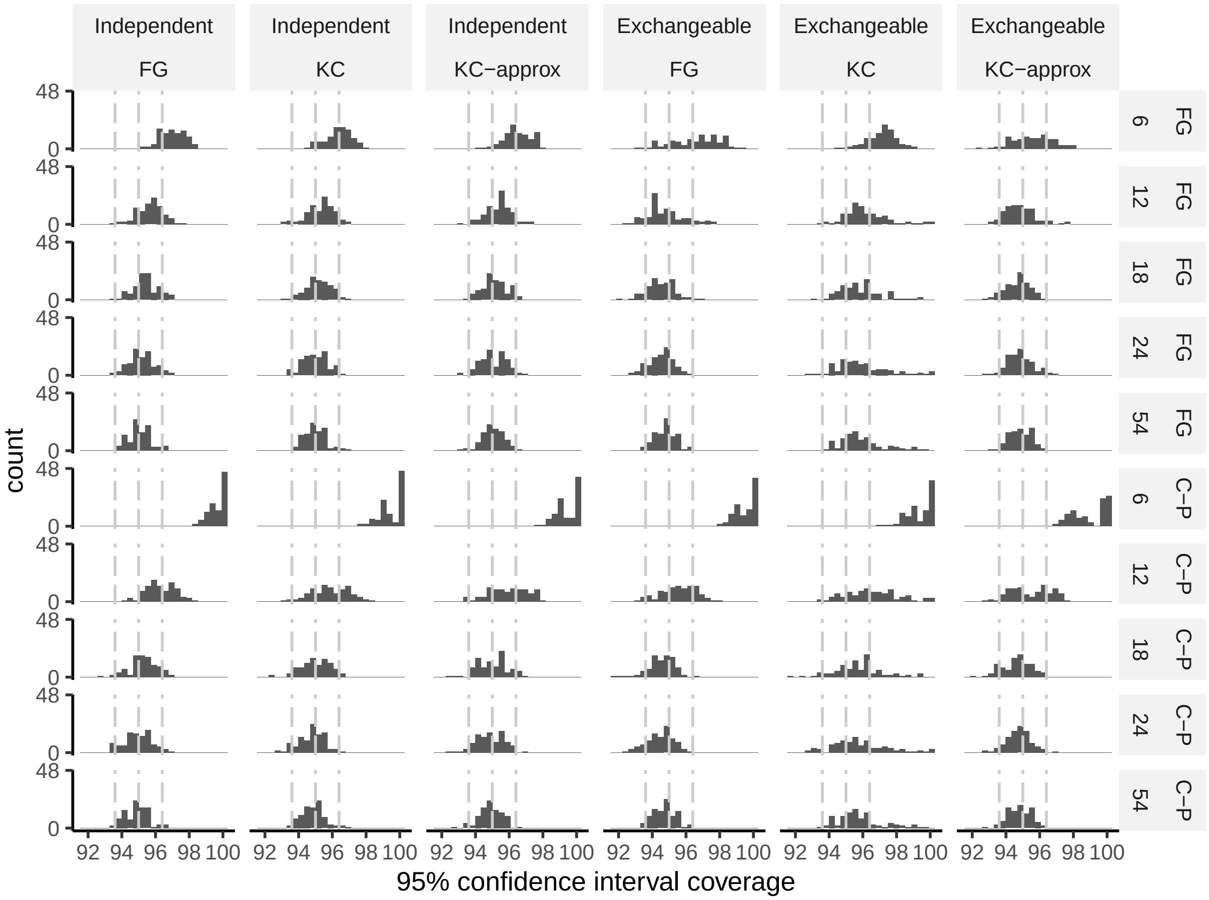

Supplementary Figure 16: Post hoc analysis: 95% confidence interval coverage with KC-approximation standard errors in phase 2 scenarios by correlation stucture

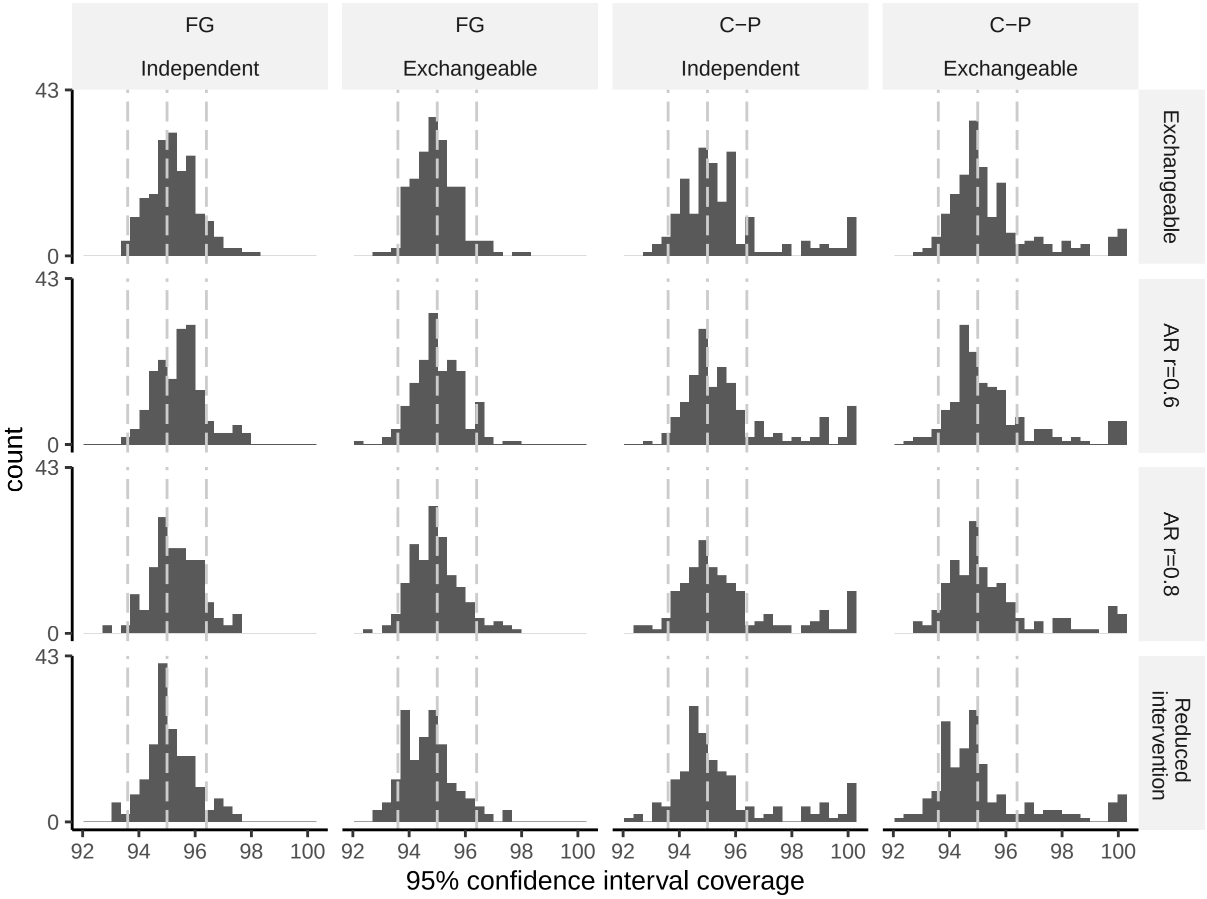

Supplement: sj-pdf-1-smm-10.1177_0962280220958735 - Supplemental material for Comparison of small-sample standard-error corrections for generalised estimating equations in stepped wedge cluster randomised trials with a binary outcome: A simulation study [file sj-pdf-1-smm-10.1177_0962280220958735.pdf]
